# Autophagy Flux Is Remodeled Sex‐ and Cell Type‐Specifically During Human Aging, and Is Linked to Reduced Physical Function in Older Adults

**DOI:** 10.1111/acel.70734

**Published:** 2026-09-27

**Authors:** Tatiana M. Moreno, Stephanie R. Heimler, Ryan J. Moran, Hava Shoshana Barkai, Lina Scandalis, Larissa Traxler, Ashley Neil, Stephen Dozier, Jaclyn Bergstrom, Alexandre R. Colas, Sanjeev S. Ranade, Anne G. Bang, Jerome Mertens, David Wing, Anthony J. Molina, Caroline Kumsta

**Affiliations:** ^1^ Graduate School of Biomedical Sciences Sanford Burnham Prebys Medical Discovery Institute La Jolla California USA; ^2^ Center for Cardiovascular and Muscular Diseases Sanford Burnham Prebys Medical Discovery Institute La Jolla California USA; ^3^ Division of Geriatrics, Gerontology, and Palliative Care, Department of Medicine University of California, San Diego La Jolla California USA; ^4^ Exercise and Physical Activity Resource Center (EPARC) University of California, San Diego La Jolla California USA; ^5^ Herbert Wertheim School of Public Health and Human Longevity Science University of California, San Diego La Jolla California USA; ^6^ Department of Neurosciences, Sanford Consortium for Regenerative Medicine University of California San Diego La Jolla California USA; ^7^ Center for Therapeutics Discovery and Conrad Prebys Center for Chemical Genomics Sanford Burnham Prebys Medical Discovery Institute La Jolla California USA

**Keywords:** aging, autophagy, direct conversion, exercise, human primary dermal fibroblasts, induced neurons (iNs), peripheral blood mononuclear cells (PBMC)

## Abstract

Autophagy is widely proposed to decline with age; however, direct evidence across human cell types remains limited. Moreover, it is unclear whether age‐associated changes in autophagy‐gene transcription are accompanied by corresponding changes in autophagic activity, and whether autophagic activity relates to physiological function during aging. We performed transcriptomic and functional autophagy analyses across subject‐matched human cell types from a healthy aging cohort. Autophagy‐related gene expression increased with age in primary dermal fibroblasts and, to a lesser extent, in induced neurons (iNs). However, autophagy flux was cell type‐ and sex‐specific and uncoupled from transcriptional remodeling. Autophagy flux decreased in male fibroblasts, remained stable in female fibroblasts, and increased in female iNs with age. In freshly isolated peripheral blood mononuclear cells (PBMCs), autophagy flux became increasingly heterogeneous with age and trended higher in older individuals, independent of sex. Associations between autophagy flux and physiological function varied across the adult lifespan; however, in adults aged > 70 years, higher autophagy flux was associated with reduced physical function. In a pilot intervention study, PBMC autophagy flux decreased following 12 weeks of mild exercise in parallel with improved physical function, suggesting that autophagic activity in PBMCs is responsive to physiological intervention in late life. Together, these findings show that autophagy is remodeled in a cell type‐, sex‐ and physiological function‐dependent manner during aging, challenge the view that autophagy uniformly declines with age, and suggest that elevated autophagy flux in older adults may reflect compensatory responses to age‐associated stress rather than enhanced autophagic capacity.

## Graphical abstract

Autophagy‐gene transcription increases with age and is uncoupled from autophagy flux. Autophagy flux is remodeled sex‐ and cell type‐specifically and is associated with worse physical function in older adults. In a pilot intervention study, elevated PBMC autophagy flux decreased following mild exercise in older adults.

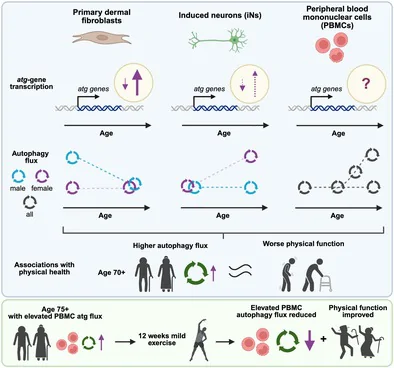

## 1 Introduction

Aging is characterized by the progressive loss of cellular and organismal health, driven by the accumulation of molecular damage, chronic stress, and impaired repair mechanisms (Lopez‐Otin et al. 2023). Among the cellular processes that maintain homeostasis during aging, macroautophagy, hereafter “autophagy,” is a conserved lysosomal degradative pathway that supports proteostasis, organelle quality control, nutrient recycling, and cellular stress adaptation (Yamamoto et al. 2023). Consistent with this central role, disabled autophagy has been identified as a primary hallmark of aging (Lopez‐Otin et al. 2023), and evidence from model organisms and post‐mortem human tissues has suggested that autophagy declines with age (Aman et al. 2021; Lim et al. 2024; Nieto‐Torres and Hansen 2021). Reduced autophagy has been linked to the pathogenesis of age‐associated diseases including neurodegenerative diseases (Nixon and Rubinsztein 2024), and interventions that enhance autophagy extend lifespan and healthspan in multiple model systems (Eisenberg et al. 2009; Hansen et al. 2018; Pyo et al. 2013).

However, autophagy is not uniformly reduced during aging. Autophagic activity can increase in specific cellular states, such as senescence and cancer (Cassidy and Narita 2022; Ebata and Hansen 2026), and transcriptional and static readouts suggest that autophagy dynamics can vary across tissues, cell types and between sexes during aging (Carosi et al. 2025). Consistent with this, recent work demonstrates cell type‐ and sex‐specific differences in chaperone‐mediated autophagy across murine tissues, although evidencing an overall trend toward decline with age (Khawaja et al. 2025). Whether age‐dependent changes occur across human cell types remains unclear, in part due to the difficulty of directly measuring autophagic activity in humans. Autophagy is a multi‐step process, and commonly used “static” readouts, including autophagosome abundance, levels of the autophagosome‐associated protein LC3/Atg8‐II, selective autophagy receptors, and autophagy‐gene expression, cannot distinguish increased autophagosome formation from impaired degradation (Moreno et al. 2025; Tanida et al. 2005). Direct measurement of autophagy flux, achieved by pharmacological inhibition of lysosomal degradation, is not feasible in vivo in humans (Klionsky et al. 2021). As a result, how autophagic activity is regulated across human cell types and physiological states during aging remains unclear.

To address these limitations, accessible human cell models that retain age‐associated features provide an opportunity to examine autophagy across cell types within the same individuals. Low‐passage primary dermal fibroblasts preserve transcriptional signatures of aging (Fleischer et al. 2018), and induced neurons (iNs) directly converted from these cells enable investigation of neuronal aging in a subject‐matched context (Mertens et al. 2021, 2015; Moreno et al. 2025; Traxler et al. 2019). In parallel, peripheral blood mononuclear cells (PBMCs) provide a minimally invasive fresh‐cell system to assess autophagy under physiologically relevant conditions (Bensalem et al. 2025, 2023; Dang et al. 2025; Singh et al. 2025). However, these approaches have not been integrated to compare transcription and functional autophagy across multiple human cell types within the same individuals.

Consequently, the relationship between autophagy‐gene transcription, autophagy flux, and physiological health in human aging remains poorly defined. It is unknown whether autophagy flux is coordinated across cell types within the same individual, whether variation in autophagy flux is associated with physiological function, and whether autophagy flux can be responsive to behavioral intervention. These questions are central to understanding whether age‐associated changes in autophagy represent a loss of functional autophagic capacity, compensatory activation, or a combination of both depending on cellular context.

Here, we performed integrated transcriptional analyses of autophagy in primary dermal fibroblasts and iNs across a healthy human cohort spanning the adult lifespan, and compared these data with autophagy flux measurements in fibroblasts, iNs, and freshly‐isolated PBMCs. We found that age‐associated changes in autophagy‐gene transcription were cell type‐specific and uncoupled from functional autophagic activity. Autophagy flux was altered in a sex‐ and cell type‐specific manner with age. We further examined associations between autophagy flux and physiological health measures, finding that higher autophagy flux was associated with reduced physical function in adults over 70 years old across all three assayed cell types. Finally, we performed a pilot intervention to test whether autophagy flux in PBMCs is modifiable by mild exercise in older adults, and found that exercise reduced elevated autophagy flux to more youthful levels. Together, these studies identify autophagy as a dynamic and context‐dependent feature of human aging that is uncoupled from transcriptional remodeling and is responsive to changes in physiological state in older adults.

## 2 Results

### 2.1 Autophagy‐Gene Transcription Is Upregulated Differently With Age in Human Primary Dermal Fibroblasts and Induced Neurons (iNs)

Aging is associated with robust transcriptomic changes in human primary dermal fibroblasts (Fleischer et al. 2018). To define age‐associated changes in autophagy‐gene transcription at a global level, we performed bulk RNA‐seq on low passage (< 10) primary dermal fibroblasts from young and older healthy adult donors (young: 23–33 years, *N* = 4; older: 64–72 years, *N* = 5) from the San Diego Nathan Shock Center Clinical Cohort (SHOCK; Table 1; Table S1), a well‐characterized cohort of healthy individuals across the age‐span who self‐reported to be free of chronic disease (Phang et al. 2024). Principal component analysis (PCA) of global fibroblast transcriptomes separated samples by age (Figure 1A), indicating retention of robust age‐associated transcriptional signatures (Fleischer et al. 2018). Differential expression analysis identified 94 significantly up‐ and 188 downregulated genes (FC > 1.0; *p*_adj_ < 0.05) in older versus younger samples (Figure S1A). Gene set enrichment analysis (GSEA) and gene ontology (GO) analysis revealed that downregulated genes were enriched for cell cycle‐related biological processes including nuclear chromosome segregation, nuclear division, and mitotic cell cycle checkpoint signaling (Figure 1B; Tables S2 and S3), consistent with reduced proliferation capacity in fibroblasts from older donors (Lago and Puzzi 2019). Consistent with senescence‐like phenotypes, MYC and EF2 target genes were negatively enriched, whereas TNFα, IL6‐JAK‐STAT3 and p53 signaling were positively enriched (Figure 1B) (Hernandez‐Segura et al. 2018; Zou et al. 2021). Upregulated genes were enriched for lipid‐associated processes including lipid localization, transport and storage (Table S2), as well as muscle‐ and endothelial‐associated terms, suggesting partial loss of cell identity with age (Salzer et al. 2018). Together, these findings validate that low‐passage SHOCK fibroblasts retain robust age‐associated transcriptomic signatures.

**TABLE 1:** Summary of San Diego Nathan Shock Center Healthy Aging (“SHOCK”) cohort demographics by experiment.

Assay	RNA‐seq		Autophagy flux		
Cell type	Fibroblast [mean (range)]	iN [mean (range)]	Fibroblast [mean (range)]	iN [mean (range)]	PBMC [mean (range)]
*N*	9	13	44	22	23
Age (years)	49.3 (23–72)	42.6 (23–76)	49.5 (23–74)	48.9 (23–74)	60.8 (24–80)
BMI (kg/m^2^)	23.30 (20.3–29.7)	24.63 (20.3–30.0)	24.64 (20.0–30.1)	24.52 (20.3–30.0)	24.51 (19.5–30.1)
Sex (% male)	44.4	46.2	52.3	63.6	47.8
Race (% White)	88.9	92.3	86.4	86.4	95.7
SPPB score	12.0 (12–12)	11.8 (10–12)	12.0 (11–12)	12.0 (11–12)	11.8 (8–12)

*Note:* SPPB, Short Physical Performance Battery, a composite measure of lower extremity function including balance, gait speed, and chair stand tests (score range 0–12, with higher scores indicating better physical function).

Abbreviations: BMI, body mass index; iN, induced neuron; PBMC, peripheral blood mononuclear cell.

**FIGURE 1:**
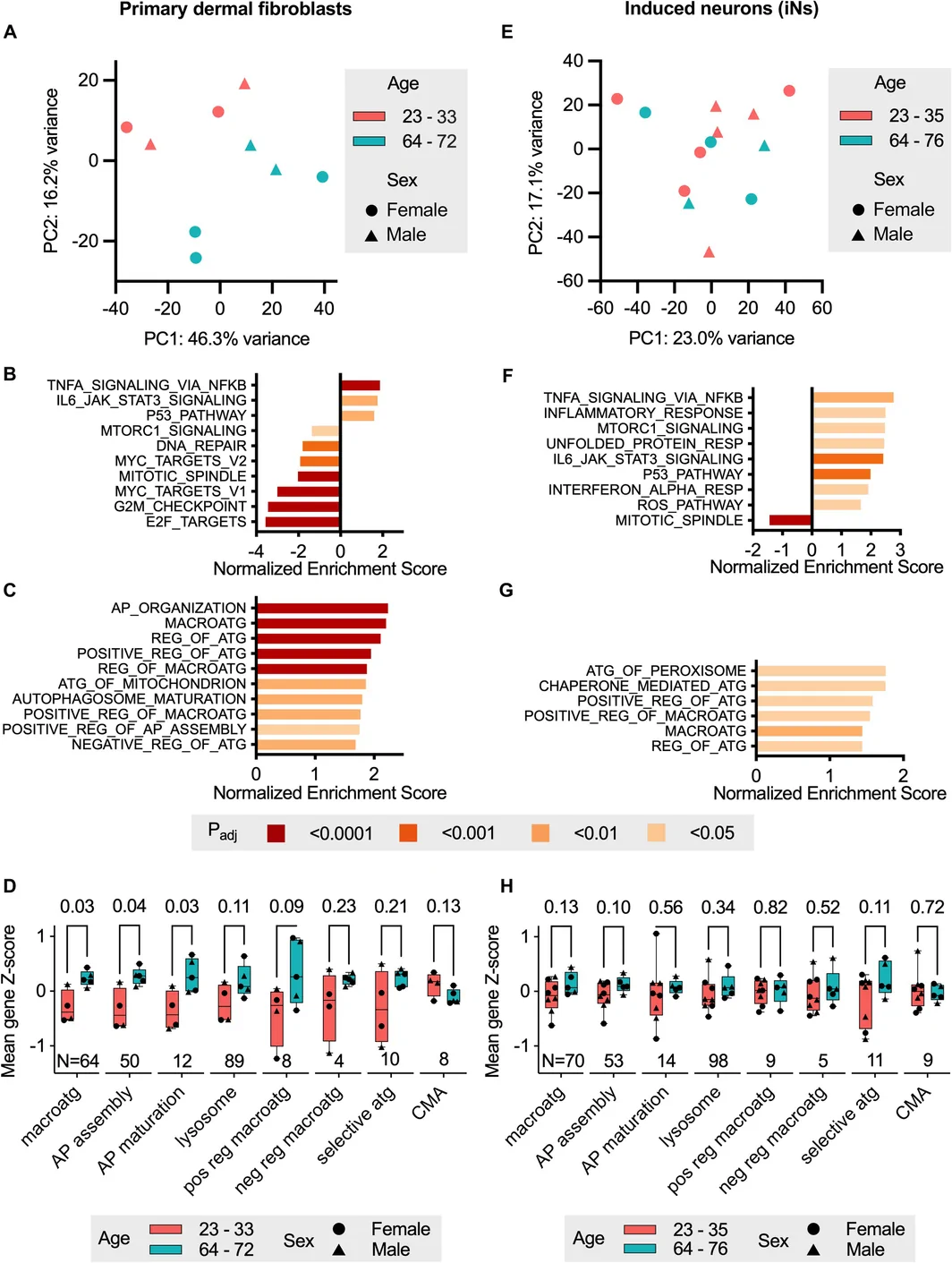
Aging is associated with upregulation of autophagy‐related transcriptional programs in primary dermal fibroblasts and iNs. (A) PCA of primary dermal fibroblast samples labeled by age group (young: 23–33 years; older: 64–72 years) and sex. (B, C) Significantly enriched (B) age‐related GSEA Hallmark and (C) autophagy‐related GO Biological Process terms in primary dermal fibroblasts from older donors. (D) Mean gene *Z*‐scores of autophagy‐related GO Biological Process gene lists in primary dermal fibroblasts from young and older donors. *p*‐values by independent *t*‐tests. (E) PCA of iN samples labeled by age group (young: 23–35 years; older: 64–76 years) and sex. (F, G) Significantly enriched (F) age‐related GSEA Hallmark and (G) autophagy‐related GO Biological Process terms in iNs from older donors. (H) Mean gene *Z*‐scores of autophagy‐related GO Biological Process gene lists in iNs from young and older donors. *p*‐values by independent *t*‐tests. For (B, C, F, G): Bars are colored by adjusted *p*‐value (*p*_adj_) as indicated in the key below panels (C, G). All significantly enriched terms (*p*_adj_ < 0.05) are listed in Tables S3 and S6. For (D, H), gene modules are listed in Table S6 and statistical comparisons are provided in Table S5. AP, autophagosome; atg, autophagy; CMA, chaperone‐mediated autophagy; macroatg, macroautophagy; neg, negative; pos, positive; reg, regulation; resp, response; ROS, reactive oxygen species. See also Figure S1.

In primary dermal fibroblasts from older donors, autophagy‐related GO Biological Process terms were positively enriched (Figure 1C). To further resolve age‐associated changes in autophagy gene transcription, we analyzed curated gene modules (Tables S4 and S5). In fibroblasts, gene modules associated with macroautophagy, autophagosome components, and autophagosome assembly and maturation were significantly upregulated with age (Figure 1D; Table S4). No clear sex‐associated patterns were detected, although this may be due to limited sample size. In contrast, other autophagy‐related modules, including selective autophagy receptors and chaperone‐mediated autophagy (CMA), were not significantly altered with age (Figure 1D). In addition, positive regulators of macroautophagy showed a trend toward upregulation (*p* = 0.09), whereas negative regulators were not significantly changed (*p* = 0.23), consistent with a regulated transcriptional response rather than stochastic age‐associated dysregulation (Figure 1D; Table S4). At the individual gene level, age‐associated changes were heterogenous: while some macroautophagy genes (e.g., *ATG5*, *ATG7*, *ATG4C*) decreased with age, consistent with prior observations (Chaves et al. 2018; Lipinski et al. 2010), the majority of macroautophagy genes increased with age or peaked in mid‐to‐late adulthood (~30–65 years; Figure S1B). Together, these findings indicate that aging in human dermal fibroblasts is associated with coordinated but gene‐specific remodeling of autophagy‐gene transcription, resulting in overall upregulation rather than a global decline.

We next sought to determine whether age‐related changes in autophagy‐gene transcription occur in a coordinated manner across human cell types, or instead show cell type‐specific remodeling. As long‐lived, post‐mitotic cells, neurons rely heavily on autophagy to maintain proteostasis, and dysfunctional autophagy has been linked to loss of proteostasis and neurodegeneration (Chou et al. 2025; Nixon and Rubinsztein 2024; Stavoe and Holzbaur 2019). However, since human neurons are typically inaccessible, especially in healthy subjects, age‐associated changes in neuronal autophagy have not been systematically characterized. We therefore assessed autophagy‐gene transcription in iNs as a model of human neuronal aging since they retain age‐associated transcriptomic signatures (Mertens et al. 2015). We generated iNs via direct conversion (Zhou‐Yang et al. 2021) of additional SHOCK donor fibroblasts, including five subject‐matched lines, and performed bulk RNA‐seq. Following conversion, iNs clustered separately from fibroblasts by PCA, confirming successful lineage conversion (Figure S1C). Although iNs showed no age‐associated transcriptomic separation by PCA analysis (Figure 1E) and had fewer differentially expressed genes (Figure S1D) compared to fibroblasts, they exhibited positive enrichment of established age‐associated pathways including inflammation, loss of proteostasis, oxidative stress and senescence (Figure 1F; Table S6) (Jurk et al. 2012; Li et al. 2023; Wodrich et al. 2022). GSEA analysis further revealed enrichment of multiple autophagy‐related pathways, including macroautophagy, and its positive regulation in aged iNs (Figure 1G; Table S6). However, in contrast to fibroblasts, autophagy gene modules were not significantly changed with age in iNs (Figure 1H), and were even more heterogenous at the individual gene level (Figure S1E). This divergence between pathway‐level enrichment and module‐level stability suggests that age‐associated changes in autophagy‐gene transcription in iNs are variable across genes and individuals, rather than reflecting a uniform, coordinated response (Figure S1E). Together, these data indicate that aging is associated with upregulation of autophagy‐related transcriptional programs in both fibroblasts and iNs, but with stronger and more coordinated upregulation in fibroblasts and more variable responses in iNs.

### 2.2 Age‐Related Changes in Autophagy Flux Are Cell Type‐ and Sex‐Specific

Given the observed increase in autophagy‐related gene transcription with age, we next asked whether these changes translated into increased autophagic activity. To address this, we measured autophagosome abundance, assessed as LC3B‐positive structures (quantified as foci area per cell area), and autophagy flux using chloroquine (CQ)‐mediated inhibition of lysosomal degradation in primary dermal fibroblasts (*N* = 44) and subject‐matched iNs (*N* = 22) from SHOCK donors. This assay allowed us to distinguish between static autophagosome abundance and functional autophagic activity. We found that static measures of LC3B‐positive foci did not reflect autophagic activity, as LC3B‐positive foci area per cell area did not correlate with autophagy flux (Figure S2A,B), demonstrating that autophagosome abundance alone is not a reliable measure of autophagic activity.

In primary dermal fibroblasts, autophagy flux exhibited sex‐specific, and age‐dependent differences. Autophagy flux in fibroblasts from young male donors trended higher than those from young female donors (Figure 2A–C), although sample size was insufficient to test sub‐group differences in autophagy flux. With increasing age, autophagy flux declined in males but remained stable in females, resulting in comparable levels between sexes in older donors (Figure 2B,C). The age‐associated decrease in autophagy flux in male donor fibroblasts was independent of total fibroblast area (Figure S3A–D). In a multiple linear regression model including an age‐by‐sex interaction, age was significantly associated with decreased autophagy flux in males (*p* = 0.021), whereas no significant age‐associated change was observed in females. The age‐by‐sex interaction showed a trend toward significance (*p* = 0.075), supporting a sex‐dependent effect of aging on autophagy flux in fibroblasts.

**FIGURE 2:**
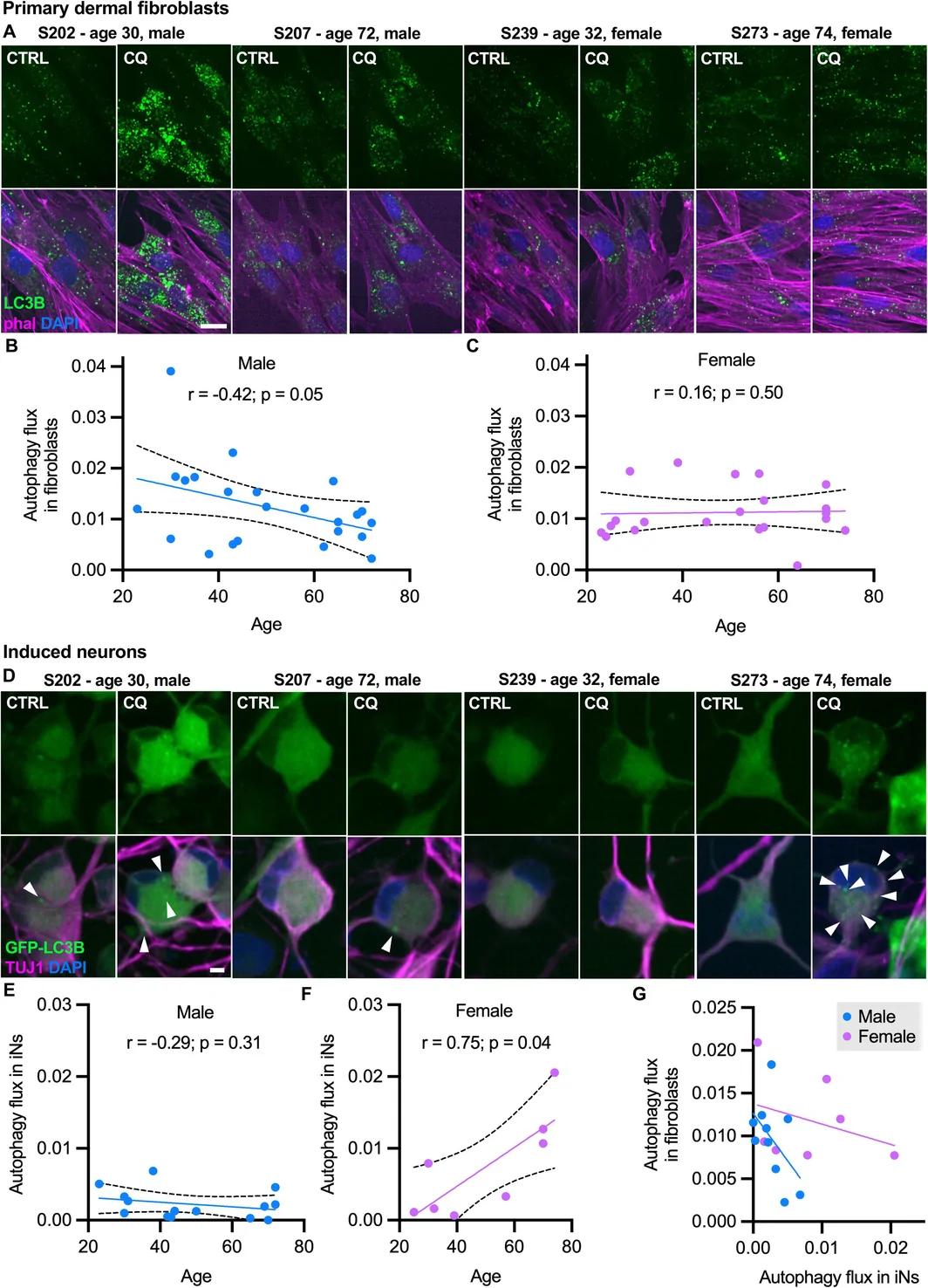
Age‐related changes in autophagy flux are cell type‐ and sex‐specific. (A) Representative images of LC3B‐positive foci (green) in primary dermal fibroblasts from young and older male (left) and female (right) donors treated ± CQ for 2 h. Actin cytoskeleton in magenta (phal—Phalloidin) and nuclei in blue (DAPI). 40× magnification. Scale bar: 20 μm. (B) Autophagy flux in primary dermal fibroblasts from male SHOCK cohort donors spanning the adult lifespan, with each point representing one donor (*N* = 23 donors) (*R* ^2^ = 0.17; *p*_slope_ = 0.05); Spearman *r* and *p*‐values reported. (C) Autophagy flux in primary dermal fibroblasts from female SHOCK cohort donors spanning the adult lifespan (*N* = 21 donors). Linear regression ±95% confidence interval shown with each point representing one donor (*R* ^2^ = 0.001; *p*_slope_ = 0.87); Spearman *r* and *p*‐values reported. (D) Representative images of GFP‐LC3B‐positive foci (green) in iNs from young and older male (left) and female (right) donors treated ± CQ for 2 h. Neuronal marker TUJ1 in magenta and nuclei in blue (DAPI). 40× magnification. Scale bar: 5 μm. (E) Autophagy flux in iNs from male SHOCK cohort donors spanning the adult lifespan (*N* = 14 donors). Linear regression ±95% confidence interval shown (*R* ^2^ = 0.08; *p*_slope_ = 0.33); Spearman *r* and *p*‐values reported. (F) Autophagy flux in iNs from female SHOCK cohort donors spanning the adult lifespan (*N* = 8 donors). Linear regression ±95% confidence interval shown (*R* ^2^ = 0.61; *p*_slope_ = 0.02); Spearman *r* and *p*‐values reported. (G) Autophagy flux in subject‐matched fibroblasts and iNs labeled by donor sex. Linear regression for males (*R* ^2^ = 0.26; *p*_slope_ = 0.14) and females (*R* ^2^ = 0.11; *p*_slope_ = 0.47) shown. For (B, C, E, G), autophagy flux reflects the absolute change in LC3B or GFP‐LC3B‐positive foci area per cell area ± CQ treatment. See also Figures S2 and S3.

In iNs, we similarly found that static measures of LC3B‐positive foci did not reflect autophagic activity, as GFP‐LC3B‐positive autophagosome area per cell area did not correlate with autophagy flux (Figure S2C,D), demonstrating that flux‐based assays are required to reliably measure autophagic activity in this cell type.

Autophagy flux in iNs also exhibited age‐ and sex‐dependent changes, although these were distinct from those observed in fibroblasts. Autophagy flux was similar between male and female cells at younger ages, remaining relatively stable with advancing age in males but increasing with age in females (Figure 2D–F). In female iNs, CQ treatment resulted in increased accumulation of GFP‐LC3B‐positive foci with increased age, consistent with increased autophagy flux (Figure S3E–H). In a multiple linear regression model, sex was significantly associated with autophagy flux (*p* = 0.016), indicating a statistically significant difference in autophagy flux between male and female samples. Inclusion of an age‐by‐sex interaction term revealed a significant interaction (*p* = 0.001), indicating that the relationship between age and autophagy flux differs by sex, with flux increasing with age in females but not in males. Together, these findings indicate that age‐related changes in autophagy flux are both cell type‐ and sex‐dependent, with opposite age‐associated trends observed in fibroblasts and iNs.

We next asked whether autophagy flux was coordinated across cell types within individuals. Autophagy flux in fibroblasts was not associated with autophagy flux in iNs across the 10 subject‐matched male samples (*r* = −0.41; *p* = 0.25) or 7 subject‐matched female samples (*r* = −0.46; *p* = 0.30) by Spearman's analysis (Figure 2G). Multiple linear regression analysis revealed a significant sex‐dependent effect on the relationship between fibroblast and iN autophagy flux (*p* = 0.02), with a stronger negative association observed in males and a weaker relationship in females. This indicates that the relationship between autophagy flux across subject‐matched cell types differs by sex and is not uniformly coordinated across cell types within individuals.

Together, these findings demonstrate that autophagy flux is regulated in a cell type‐ and sex‐dependent manner during human aging and does not mirror transcriptional changes in autophagy‐related genes. This uncoupling highlights the importance of directly measuring autophagy flux to accurately assess autophagic activity.

### 2.3 Autophagy Flux Becomes More Heterogeneous With Age in Freshly Isolated Human PBMCs

Given the tissue‐ and sex‐specific regulation of autophagy flux in fibroblasts and iNs, we next assessed age‐associated changes in autophagy flux in freshly isolated PBMCs from SHOCK cohort participants (Figure 3A). Whole‐blood and isolated‐cell Coulter counts showed that our isolation method strongly enriched for lymphocytes (Figure S4A), indicating that measured autophagy flux primarily reflected activity in T‐, B‐, and NK cells. Technical variability across CQ treatment conditions was low for lymphocytes (CV < 20%) and increased only in scarce cell types (Figure S4B), indicating consistent PBMC composition across samples.

**FIGURE 3:**
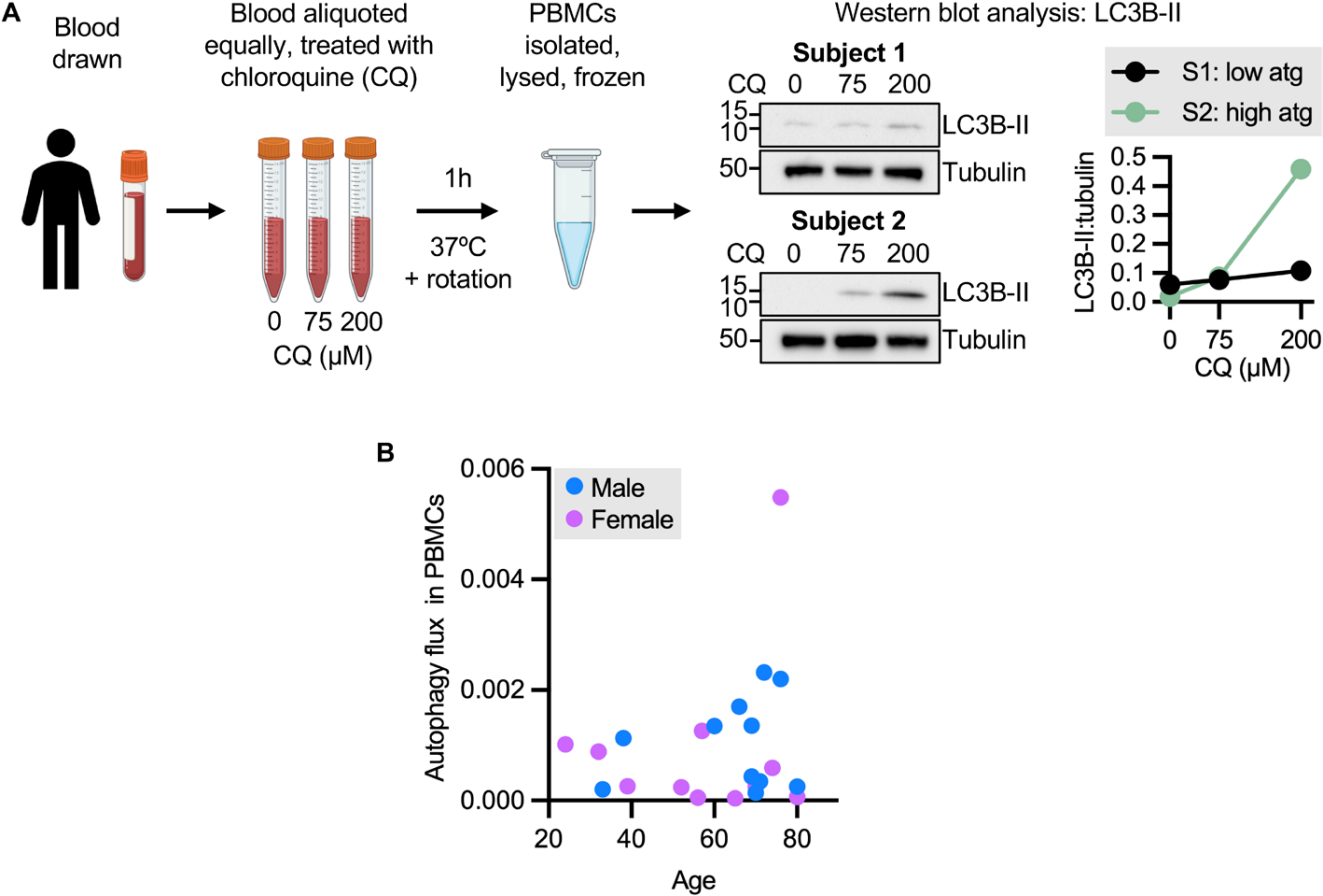
Autophagy flux becomes more heterogeneous with age in freshly isolated human PBMCs. (A) Experimental schematic of the autophagy flux assay in freshly drawn whole blood, followed by PBMC isolation and Western blot analysis of autophagosome marker LC3B‐II. (B) Autophagy flux in PBMCs from male (*N* = 11) and female (*N* = 12) SHOCK cohort donors spanning the adult lifespan, with each point representing one donor. See also Figures S4–S6.

As in fibroblasts and iNs, basal LC3B‐II levels in untreated samples did not correlate with autophagic flux (Figure S5A), whereas LC3B‐II levels following CQ treatment correlated strongly with measured flux (Figure S5B), confirming the requirement for flux‐based assays in PBMCs.

Autophagy flux in PBMCs from 23 SHOCK cohort participants was not associated with age (*p* = 0.44); however, autophagy flux became more heterogeneous with increasing age and showed a trend toward higher flux in individuals age ~70 and above (Figure 3B). This pattern is consistent with previous reports of increased autophagy flux with age in freshly isolated PBMCs (Bensalem et al. 2023; Dang et al. 2025). No sex‐associated differences in PBMC autophagy flux were observed (multiple linear regression main effects *p* = 0.86; age:sex interaction *p* = 0.91), indicating that PBMC autophagy flux is not significantly influenced by sex in this cohort. We further confirmed that these findings were not explained by age‐related differences in PBMC composition, either in whole blood or after isolation (Figure S6A,B). Thus, unlike fibroblasts and iNs, PBMC autophagy flux exhibited increased heterogeneity with age but no detectable sex‐specific effects.

These data further support a model in which autophagic activity is not coordinately regulated across cell types within individuals. Instead, fibroblasts, iNs, and PBMCs exhibited distinct age‐associated flux patterns, reinforcing cell‐type specificity as a central feature of autophagy regulation during human aging.

### 2.4 Increased Autophagy Flux in Human Cells Correlates With Reduced Physical Fitness in Older Adults

We next asked whether autophagy flux in primary dermal fibroblasts, iNs, and PBMCs could be indicative of physical health and fitness. To assess organismal fitness, SHOCK cohort participants underwent standardized clinical and functional assessments, performed at rest and during maximal exertion during progressive treadmill exercise (VO_2max_) (Phang et al. 2024). Measures of exercise capacity, metabolic function and physical performance declined with age in the SHOCK healthy aging cohort, including resting VO_2_ (Figure S7A), maximum heart rate (Figure S7B), VO_2max_ (Figure S7C), minute ventilation (Figure S7D), grip strength (Figure S7E), time spent in the very vigorous activity zone as measured by actigraphy (age‐associated decline strongly driven by extremely high values in two young participants) (Figure S7F), and walking speed (Figure S7G), consistent with well‐established age‐related reductions in aerobic capacity, muscle function, and high‐intensity physical activity (Cooper et al. 2010; Letnes et al. 2023; Troiano et al. 2008). In contrast, measures of cardiovascular load and decreased physical function increased with age, including systolic and diastolic blood pressure (Figure S7H,I), and chair stand time (Figure S7J), reflecting known age‐associated increases in vascular stiffness and cardiovascular risk (Laurent and Boutouyrie 2020), and decreased mobility (Huisingh‐Scheetz et al. 2021). Notably, a subset of measures showed little or no association with age, including body‐mass‐index (BMI) (Figure S7K), resting heart rate (Figure S7L), Short Physical Performance Battery (SPPB) score (a composite measure of lower extremity function including balance, gait speed, and chair stand tests) (Figure S7M), and accelerometer‐derived sedentary time (Figure S7N), indicating preservation of baseline functional status in this cohort.

We next examined how these physiological measures relate to autophagy flux across cell types. Across SHOCK cohort participants, autophagy flux showed distinct associations with physiological outcomes that differed by cell type. In fibroblasts and PBMCs, higher autophagy flux correlated with measures indicative of more youthful cardiovascular function, including higher maximum heart rate (fibroblasts: Spearman *r* = 0.27; *p* = 0.075) (Tanaka et al. 2001) and lower resting heart rate (PBMCs: Spearman *r* = −0.61; *p* = 0.002) (Gonzales et al. 2023) (Figure 4A). In contrast, in iNs, higher autophagy flux correlated with reduced maximal grip strength (Spearman *r* = −0.51; *p* = 0.016) and walking speed (Spearman *r* = −0.52; *p* = 0.01) (Figure 4A), suggesting that increased autophagy flux in iNs may be associated with reduced physical function (Cooper et al. 2010). Together, these findings indicate that autophagy flux is not simply a readout of chronological aging, but instead reflects aspects of physiological health that vary across individual and cell type.

**FIGURE 4:**
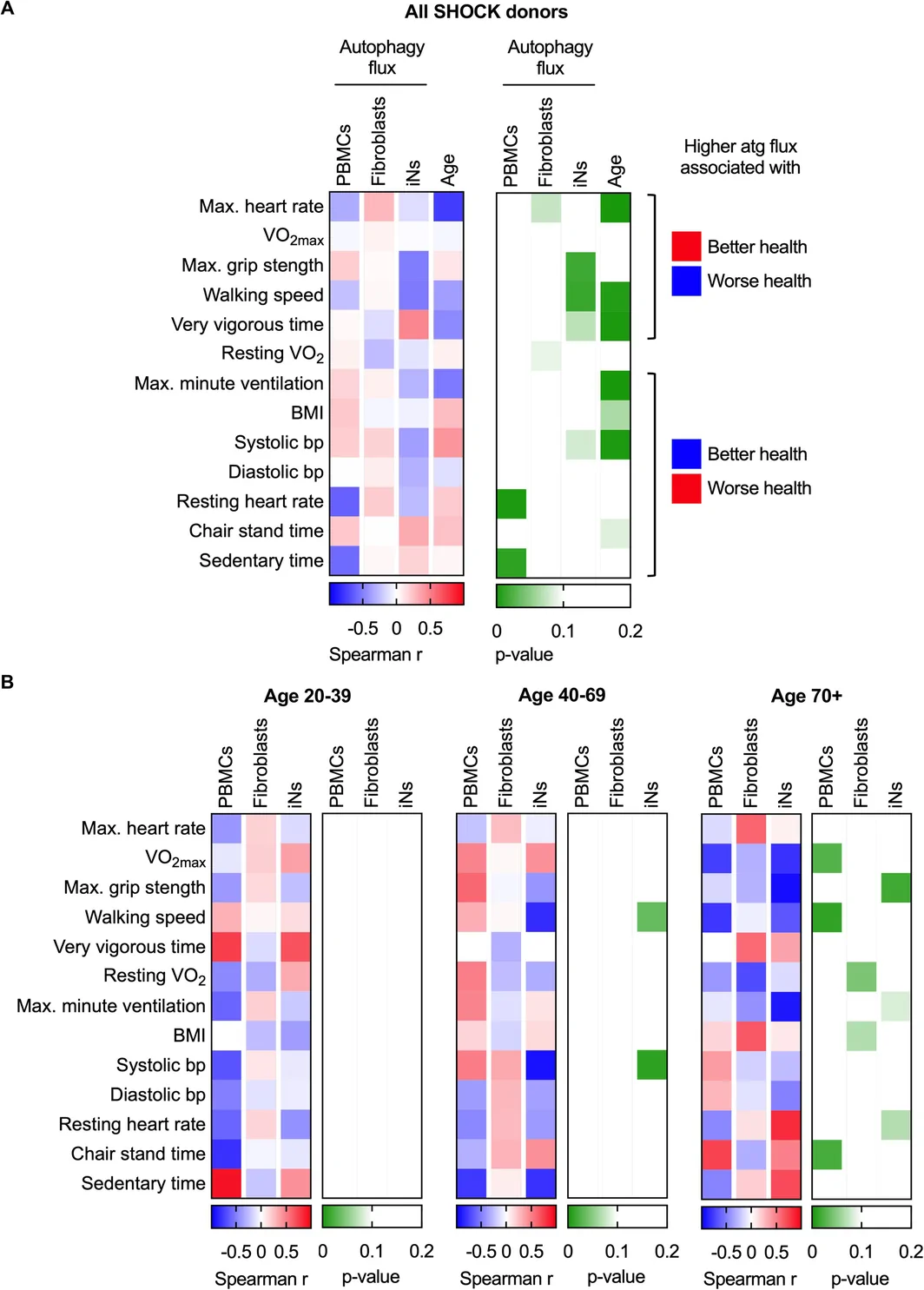
Increased autophagy flux in human cells correlates with reduced physical fitness in older adults. Heatmaps showing Spearman correlation coefficients (*r*) and corresponding *p*‐values between autophagy flux in PBMCs, primary dermal fibroblasts or iNs, as well as age and physiological health readouts. Data are shown in (A) all SHOCK cohort participants, and (B) SHOCK cohort participants stratified by age group. In (A), the annotation on the right indicates whether higher values of each physiological measure are associated with better or worse health. Accordingly, positive correlations between autophagy flux and measures associated with better health indicate improved fitness, whereas positive correlations with measures associated with worse health indicate reduced fitness, and vice versa for negative correlations. Spearman correlations were calculated separately for each cell type and visualized as heatmaps. For all SHOCK cohort participants shown in (A), PBMCs, *N* = 23; fibroblasts, *N* = 44; iNs, *N* = 22; Age, *N* = 54. For SHOCK cohort participants in (B), Age 20–39: PBMCs, *N* = 5; fibroblasts, *N* = 15; iNs, *N* = 9; age 40–69: PBMCs, *N* = 8; fibroblasts, *N* = 20; iNs, *N* = 7; age 70+: PBMCs, *N* = 10; fibroblasts, *N* = 9; iNs, *N* = 6. See also Figure S7.

Since autophagy flux became more heterogeneous in PBMCs in adults aged approximately 70 and older, and maximum autophagy flux in these subjects was relatively higher than in younger subjects (Figure 3B), we next asked whether these relationships differed at advanced ages. Indeed, in SHOCK participants aged 70 years and older, higher autophagy flux across cell types was more consistently associated with reduced physical fitness and poorer performance on frailty‐related functional measures (Clegg et al. 2013). Specifically, higher PBMC autophagy flux was associated with lower VO_2max_ (Spearman *r* = −0.75; *p* = 0.026), slower walking speed (Spearman *r* = −0.78; *p* = 0.01) and longer time to complete the chair stand test (Spearman *r* = 0.73; *p* = 0.02); higher fibroblast autophagy flux correlated with increased BMI (Spearman *r* = 0.65; *p* = 0.067) and reduced resting VO_2_ (Spearman *r* = −0.70; *p* = 0.043); and higher iN autophagy flux was associated with reduced maximal grip strength (Spearman *r* = −0.94; *p* = 0.017), and increased resting heart rate (Spearman *r* = 0.82; *p* = 0.067) (Figure 4B), although these relationships should be interpreted cautiously given the small sample size for these outcomes. With the exception of resting VO_2_, which is not indicative of health status on its own (Leal‐Martin et al. 2022), these measures are broadly consistent with reduced physical fitness and poorer performance on frailty‐related functional assessments, although frailty itself was not directly measured using a validated frailty index. Together, these findings suggest that elevated autophagy flux may have different biological implications in younger and older adults.

Together, these results indicate that the relationship between autophagy flux and physiological health differs with age. While higher autophagy flux shows variable associations with fitness across the adult lifespan depending on cell type, in older adults higher autophagy flux is consistently associated with reduced physical fitness. Thus, high autophagy flux should not be interpreted as uniformly beneficial or detrimental without considering age and cell type.

### 2.5 Mild Exercise Reverses Elevated Autophagy Flux in Fresh PBMCs

To test whether PBMC autophagy flux is modifiable in older adults with reduced physical function, we measured autophagy flux in participants from the Strong Foundations 2.5 (STRONG) cohort, a digitally delivered fall‐prevention exercise program for older adults (Wing et al. 2025) (Table 2; Table S7). STRONG participants completed a 12‐week mild exercise program consisting of weekly 1‐hour sessions focused on improving balance, muscular strength and posture. Health and fitness measures were collected before and after the 12‐week intervention program (Figure S8), and included measures that overlapped with those assessed in the SHOCK healthy aging cohort, such as BMI, SPPB, maximal grip strength, resting heart rate, and resting systolic and diastolic blood pressure (Figure S9). Maximal exertion testing was not performed in STRONG participants. This pilot study included STRONG participants aged 77–88 (Figure S9A; Table S7). SPPB scores in the STRONG cohort were significantly lower than in the SHOCK cohort, indicating poorer functional capacity in STRONG participants (Figure S9C; Table S8).

**TABLE 2:** Summary of STRONG participant demographics and fitness measures before and after a 12‐week mild exercise intervention.

	Baseline, Week 0 [mean (range)]	12‐weeks mild exercise [mean (range)]
*N*	5	
Age (years)	83.8 (77–88)	
Sex (% male)	60.0	
Race (% White)	100.0	
BMI (kg/m^2^)	27.5 (23.9–31.6)	29.0 (24.6–36.7)
SPPB score	8.6 (6–11)	9.8 (8–12)

*Note:* SPPB, Short Physical Performance Battery, a composite measure of lower extremity function including balance, gait speed, and chair stand tests (score range 0–12, with higher scores indicating better physical function). All measurements were obtained from the same participants at baseline (Week 0) and after 12 weeks of exercise.

Abbreviation: BMI, body mass index.

Baseline PBMC autophagy flux measurement in STRONG cohort participants revealed elevated flux relative to the SHOCK cohort mean, with 4 of 5 STRONG participants exceeding one standard deviation above the SHOCK cohort average across all ages (Figure 5A). These findings strengthen the observation that higher PBMC autophagy flux in older adults is associated with reduced physical function.

**FIGURE 5:**
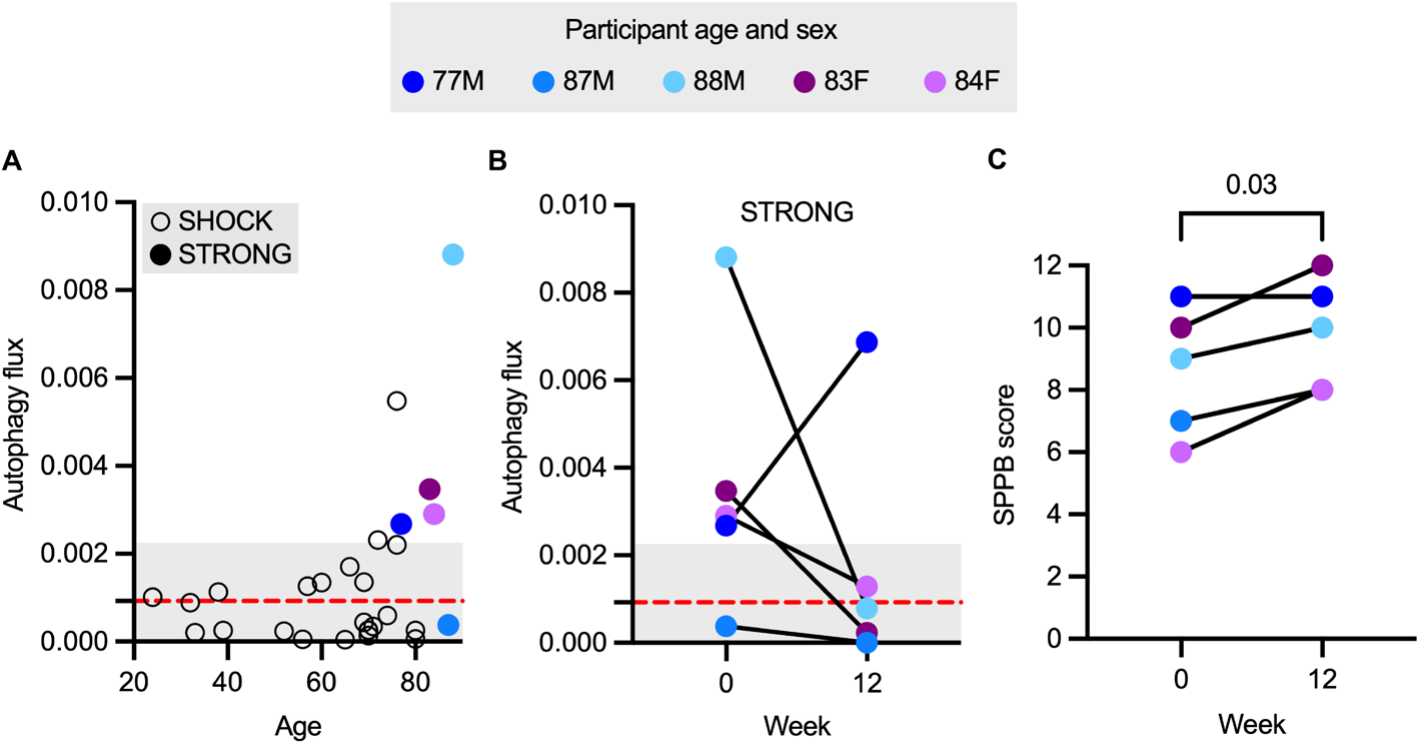
Mild exercise is associated with reduced PBMC autophagy flux and improved physical performance in older adults. Data points for STRONG cohort participant are colored by age (years) and sex (F, female; M, male). (A) Association between age and autophagy flux in PBMCs from SHOCK cohort participants (open circles; *N* = 23) and STRONG cohort participants at baseline (colored circles; *N* = 5). (B) Autophagy flux in STRONG cohort participants before and after a 12‐week mild exercise program (*N* = 5). Each line represents one participant. *p*‐value by paired t‐test. Red dotted line and gray shading in (A) and (B) indicate mean SHOCK cohort autophagy flux ± standard deviation. (C) SPPB score in STRONG cohort participants before and after a 12‐week mild exercise program. *p*‐value by paired *t*‐test. See also Figures S8 and S9.

Following the 12‐week mild exercise program, PBMC autophagy flux decreased in 4 out of 5 participants and fell within one standard deviation of the SHOCK Healthy Aging cohort mean (Figure 5B). In parallel, SPPB scores improved in STRONG subjects (Figure 5C), while BMI (Figure S8A) and resting heart rate (Figure S8B) were mildly increased, and maximal grip strength (Figure S8C), posture (Figure S8D,E), blood pressure (Figure S8F,G), and chair stands (Figure S8H; Tables S7 and S8) were unchanged. These changes were not accompanied by significant alterations in PBMC composition in either whole blood or isolated PBMC preparations (Figure S10), suggesting that the reduction in autophagy flux was not driven by shifts in major immune cell populations.

These pilot data suggest that mild exercise may reduce PBMC autophagic activity while improving physical performance in older adults, indicating that PBMC autophagy flux remains responsive to physiological intervention in late life.

Interestingly, the single participant in whom autophagy flux increased following the intervention was the youngest (age 77) and the most physically functional at baseline (SPPB score = 11), whereas all other participants were age 83 or older. Although limited by sample size, this observation is consistent with the possibility that the relationship between PBMC autophagy flux and physiological fitness state may differ at more advanced ages. Overall, these preliminary findings indicate that autophagy flux in circulating immune cells is modifiable in humans and further support an age‐dependent relationship between autophagy flux and physical function.

## 3 Discussion

In this study, we identified cell type‐ and sex‐specific differences in how autophagy flux changes with age in humans across PBMCs and subject‐matched cultured primary dermal fibroblasts and iNs, and show that these functional changes are uncoupled from autophagy‐related gene transcription. We further show that autophagy flux is associated with measures of physical function and fitness, and is responsive to mild exercise in older adults. These findings challenge the prevailing view that autophagy uniformly declines with age and instead support a model in which autophagy is remodeled in a context‐dependent manner across cell types, sexes, and physiological states. Moreover, our data suggest that elevated autophagy flux may have distinct biological implications across the lifespan.

In primary dermal fibroblasts, we find that autophagy‐related gene expression is broadly upregulated with age, with enrichment of pathways associated with macroautophagy and lysosomal function, while modules associated with selective autophagy receptors and chaperone‐mediated autophagy were not significantly altered. This suggests macroautophagy‐specific transcriptional remodeling with heterogeneous changes on the individual gene level (Chaves et al. 2018; Lipinski et al. 2010). This transcriptional upregulation may reflect a compensatory response to age‐associated cellular stress and accumulation of autophagic substrates, potentially driven by activation of stress‐responsive regulators of autophagy such as transcription factor EB (TFEB) or forkhead box O (FOXO) (Kuo et al. 2025; Moreno et al. 2022). Despite this overall transcriptional upregulation, these changes did not translate into uniformly increased functional autophagic activity in human dermal fibroblasts. Instead, our data indicate that age‐associated changes in autophagy flux in fibroblasts are sex‐dependent, with a decline observed in males and relative stability in females. Whether the age‐associated decline in males reflects loss of adaptive capacity, reduced demand for autophagy, or altered lysosomal function remains unclear and will require direct assessment of autophagosome quality and lysosomal function.

In iNs, autophagy flux exhibited distinct age‐ and sex‐dependent patterns that differed from those observed in fibroblasts, including an increase in flux with age in females and relative stability in males. These findings indicate that neuronal autophagy is regulated in a cell type‐ and sex‐specific manner and, when compared with subject‐matched fibroblasts, further suggest that autophagy is not coordinately regulated across cell types within individuals. At the transcriptional level, aging in iNs was associated with enrichment of autophagy‐related pathways, including macroautophagy and its positive regulation; however, in contrast to fibroblasts, autophagy gene modules were not significantly altered with age, indicating a less coordinated transcriptional response. Future studies comparing autophagy gene transcription and flux in subject‐matched directly converted and iPSC‐derived neurons will additionally help clarify how conversion strategy influences autophagy regulation and strengthen the generalizability of these findings across neuronal models. Although directly converted neurons retain age‐associated molecular signatures, they remain an in vitro model and may not fully recapitulate the in vivo neuronal environment, including exposure to circulating factors, neuronal circuitry, and tissue‐specific architecture. Moreover, given that autophagy is altered at the proteomic and functional levels in iNs derived from patients with neurodegenerative diseases (Chou et al. 2025; Drouin‐Ouellet et al. 2022; Pircs et al. 2022), it will be important to map these changes longitudinally to better understand how autophagy evolves during the transition from healthy aging to disease states.

These findings also raise the question of how age‐associated autophagy changes relate to shifts in cellular state. Both fibroblasts and iNs showed enrichment of senescence‐related pathways with age, suggesting the emergence of senescence‐associated or senescence‐like transcriptional programs. However, only fibroblasts showed significant age‐associated upregulation in macroautophagy‐ and lysosome‐related transcriptional modules. Since these analyses were performed on bulk cultures, we cannot distinguish whether these transcriptional changes reflect remodeling within aged but non‐senescent cells, an increased abundance of senescent or senescent‐like cells, or both. Future studies integrating autophagy flux measurements with senescence marker analysis will be required to distinguish between these possibilities.

Taken together, our data reveal a disconnect between transcriptional and functional measures of autophagy, highlighting that similar transcriptional upregulation across cell types can be associated with divergent functional outcomes. This underscores the importance of directly measuring autophagy flux rather than relying on transcriptional proxies, and points to post‐transcriptional and post‐translational mechanisms as regulators of autophagic activity during human aging. Defining these regulatory layers will be critical for distinguishing changes in autophagic capacity from changes in autophagy demand, and future studies integrating transcriptomic with proteomic analyses will be important for determining the mechanism by which aging modulates the autophagy pathway. Importantly, because autophagic activity was assessed using LC3B‐based flux assays, our study cannot distinguish changes in cargo selectivity, autophagosome quality, autophagosome maturation, or lysosomal competence, all of which may contribute to age‐associated alterations in autophagic function. Integrating flux measurements with complementary assessments of lysosomal activity and cargo degradation will therefore be important for defining the mechanisms underlying age‐associated autophagy remodeling.

Recent studies have begun to measure autophagy flux in freshly isolated PBMCs, demonstrating that autophagy flux is heterogeneous and can increase with age (Bensalem et al. 2023; Dang et al. 2025). Consistent with these observations, our data show that autophagy flux in PBMCs becomes increasingly heterogeneous with age and trends higher in older individuals. Importantly, we find that elevated autophagy flux in older adults is associated with reduced physical function and fitness. While dietary interventions including caloric restriction, intermittent fasting and acute protein intake (Bensalem et al. 2025; Singh et al. 2025, 2026) do not significantly modulate of autophagy flux in freshly isolated PBMCs, our pilot intervention of mild exercise over a 12‐week period induced measurable changes in autophagy flux in older adults, reducing autophagy flux toward levels observed in younger individuals. These data support a role for exercise as a distinct modulator of autophagic activity in circulating immune cells, unlikely to be confounded by dietary variability.

Since PBMCs are a heterogeneous mixture of immune cell types, future studies should determine whether age‐ and exercise‐associated changes in PBMC autophagy flux are shared across major PBMC classes or driven by specific subpopulations. Recent work showed that basal autophagy flux is similar across the major PBMC classes and correlates well with overall PBMC autophagy flux, although differences were observed between monocyte subsets and in response to nutrient restriction (Dang et al. 2025). These data support the use of bulk PBMC measurements under basal conditions. Nevertheless, PBMC subtype‐specific autophagy flux assays and single‐cell approaches will be important for defining how aging and exercise remodel autophagy across circulating immune‐cell populations.

Across the adult lifespan in our cohorts, higher autophagy flux was not uniformly associated with poorer physiology and in some contexts correlated with measures of more youthful cardiovascular function. In contrast, elevated flux in older individuals was more consistently associated with reduced physical performance, including poorer performance in frailty‐related functional measures. Although limited by the small cohort size and restricted sampling timepoints, the parallel reduction in PBMC autophagy flux and improvement in physical performance suggest that elevated autophagy flux in older adults may not necessarily indicate enhanced autophagic capacity. Rather, elevated autophagy flux in older adults may represent increased autophagy demand or compensatory responses to cellular stress, whereas high autophagy flux in younger individuals may reflect greater basal autophagic capacity. This distinction has important implications for the development of pharmacological or dietary strategies aimed at enhancing autophagy to promote healthy aging. To this end, future studies in larger cohorts should incorporate additional readouts of biological aging, including frailty and cognitive measures, to better stratify individuals according to biological rather than chronological age, to define the biological basis of elevated autophagy flux in younger and older individuals, and determine when increased autophagy flux is physiologically beneficial.

## 4 Methods

### 4.1 SHOCK Cohort for Primary Dermal Fibroblast, Induced Neuron, and Peripheral Blood Mononuclear Cell Studies

The San Diego Nathan Shock Center Clinical Cohort (SHOCK) has previously been described (Phang et al. 2024), including standardized assessments of physical and cognitive performance. All participants provided informed consent, and all procedures were approved by University of California, San Diego (UC San Diego) IRB 201141. For the present study, participant inclusion criteria differed for each experimental approach (Table 1). For RNA‐seq analyses, primary dermal fibroblast and directly converted iN donor lines were selected a priori to represent relatively younger and older male and female SHOCK participants. For autophagy flux analyses, primary dermal fibroblast analyses included all cell lines available to our laboratory at the beginning of the study, whereas fibroblast lines for iN experiments were selected a priori to span the participant age range while including both male and female donors. PBMC samples were obtained from participants enrolled after implementation of the PBMC autophagy flux protocol and IRB amendment, for whom fresh blood collection and immediate processing by trained study personnel were feasible. Physical health measures for all SHOCK participants were collected, as previously described (Phang et al. 2024). Autophagy flux measurements in PBMCs were correlated with participant age, sex, and physiological fitness measures obtained under resting conditions and following exercise to volitional exhaustion. Additional experimental, demographic, and health data for each participant are available in Table S1.

### 4.2 STRONG Cohort for PBMC Studies Before and After 12 Weeks of Mild Exercise

The Strong Foundations 2.5 (STRONG) cohort sponsored by UCSD's Exercise and Physical Activity Resource Center underwent a similar intervention to a Strong Foundations cohort that has previously been described (Wing et al. 2025), including standardized assessments of physical and cognitive performance. All participants provided informed consent, and all procedures were approved by UC San Diego's IRB 809304. Participants were residents of senior living facilities in San Diego, CA, identified as low‐ or moderate‐risk for falls, who volunteered to participate in a modified, partially digitally delivered exercise program designed to improve strength and prevent falls. For the present study, the IRB protocol was amended to include PBMC autophagy flux measurements before and after the 12‐week exercise intervention. Participants were included when fresh blood collection and immediate processing by trained study personnel were feasible at both study visits. Consequently, paired baseline and post‐intervention PBMC autophagy flux measurements were available for five participants. Although more limited in scope than the foundational study, the methodology for the physical health and performance metrics for all STRONG participants collected at baseline (Week 0) and post‐intervention (Week 12) timepoints has been previously described (Wing et al. 2025) (Table S7). Changes in autophagy flux in PBMCs and corresponding changes in physical fitness between baseline (Week 0) and post‐intervention (Week 12) were analyzed (Table S8).

### 4.3 Cell Culture

Primary dermal fibroblasts from donors aged 23–76 years were obtained from the San Diego Nathan Shock Center (SD‐NSC). Cells were cultured at 37°C, 5% CO_2_ and ambient O_2_ (~21%) in DMEM (Gibco #11965092) supplemented with 15% fetal bovine serum (FBS, Corning), 1% non‐essential amino acids (NEAA, Gibco #11140050), and 1% GlutaMAX (Gibco #35050061), referred to as “fibroblast media.” All primary dermal fibroblast lines were at or below passage 12 for all experiments (Table S1).

### 4.4 Lentivirus Production and Titration

Production of UNA lentivirus for fibroblast to iN conversion was performed as previously described (Zhou‐Yang et al. 2021). Briefly, HEK293T cells were transfected with pLVXUbC‐rtTA‐Ngn2:2A:Ascl1 (Addgene: #127289), and the packaging plasmids psPAX2 (Addgene #12260) and pMD2.G (Addgene #12259) using PEI. Viral titers were quantified using Lenti‐X GoStix Plus (Takara) and concentrated by ultracentrifugation after 72 h of incubation. Concentrated virus was resuspended in DPBS and stored at −80°C.

For production of EGFP‐LC3B lentivirus, large‐scale lentivirus production and concentration were performed by the Sanford Burnham Prebys Functional Genomics core facility. The lentivirus production procedure and conditions were optimized using Biomek i7 automation at high‐throughput scale and subsequently scaled up to 150 cm^2^ culture dishes for larger‐scale production (Yang et al. 2025). Briefly, pCDH‐EF1a‐mCherry‐EGFP‐LC3B lentivector DNA (Addgene #170446) was co‐transfected with psPAX2 (Addgene #12260) and pMD2.G (Addgene #12259) plasmids into HEK293T cells in nine 150 cm^2^ dishes. Viral supernatant was collected every 24 h from Day 2 to Day 4 post‐transfection. Crude virus was filtered through a 0.22 μm filter and concentrated by ultracentrifugation at 21,000 rpm for 2 h at 4°C using a 20% sucrose gradient. Concentrated viral particles were resuspended in DPBS, aliquoted, and stored at −80°C. Viral titers were determined by measuring transduction efficiency in target cells using a LSRFortessa (BD) flow cytometer at 4 days post‐transduction.

### 4.5 Generation of Directly‐Reprogrammed Neurons From Primary Dermal Fibroblasts

Induced neurons (iNs) were generated from SHOCK cohort primary dermal fibroblasts, as previously described (Zhou‐Yang et al. 2021). Briefly, primary dermal fibroblasts were transduced with lentivirus pLVXUbC‐rtTA‐Ngn2:2A:Ascl1 (Addgene #127289) carrying puromycin resistance and doxycycline‐inducible neuronal transcription factors NGN2 and ASCL1 (UNA cassette). Transduced cells were expanded under puromycin selection (1 μg/mL) in TFM‐P media (Zhou‐Yang et al. 2021) for at least five passages. Primary dermal fibroblasts carrying the UNA cassette are referred to as “UNA+ fibroblasts.” UNA+ fibroblasts were generated either in‐house or obtained from the SD‐NSC. All UNA+ fibroblast lines were maintained below passage 18.12, meaning 18 passages from fibroblast isolation and 12 passages after transfection, at the time of conversion (Table S1). For iN conversion, UNA+ fibroblasts were pooled to 300% confluency in TFM‐P, and after 24 h, changed to iN conversion media (referred to as “NK media”), containing doxycycline and a defined combination of neuronal conversion factors (Zhou‐Yang et al. 2021). NK media was replaced every 2 days for the first week, followed by three times per week thereafter. iNs were harvested on Day 21 post‐induction for RNA‐seq analysis, or purified on Day 23 post‐induction for autophagy flux experiments.

### 4.6 RNA‐Seq Analysis of Primary Dermal Fibroblasts and iNs

Primary human dermal fibroblasts were selected a priori to represent relatively younger (age < 35) and older (age > 60) male and female donors. Cultures from 4 young (23–33 years) and 5 older (64–72 years) SHOCK cohort donors were analyzed (see Table 1; Table S1). Cells were cultured in fibroblast media. For RNA‐seq, 200,000 cells per culture were seeded in single wells of 6‐well plates and grown under the previously described conditions for 48 h. RNA was extracted following the RNeasy RNA purification kit protocol (Qiagen #74104). Cells were washed with Dulbecco's phosphate‐buffered saline (DPBS, Gibco # 14190094) and lysed in RNeasy lysis buffer containing β‐mercaptoethanol, followed by homogenization using QIAshredder columns (Qiagen #79656), and purification with RNeasy Mini spin columns, including on‐column DNase digestion. PolyA library prep was performed by the Salk Institute for Biological Studies Next Generation Sequencing and Genomics Core using the Illumina NovaSeq 6000 SP to generate paired‐end reads (50 bp) at an average depth of ~50 million reads per sample.

Directly converted iNs were generated from a predefined subset of relatively younger and older male and female SHOCK cohort donors. iNs directly converted from UNA+ fibroblasts from 8 young (23–35 years) and 5 older (64–76 years) SHOCK cohort donors were analyzed. After 3 weeks of conversion, iNs were detached using TrypLE and purified by magnetic‐associated cell sorting (MACS) using PSA‐NCAM antibody‐associated beads (Miltenyi cat# 130‐092‐966) over MACS columns (Miltenyi cat# 130‐042‐201). 200,000–300,000 purified iNs per sample were pelleted and snap‐frozen. RNA was extracted using the TRIzol LS protocol, and genomic DNA was removed using TURBO DNase (Thermo Fisher Scientific). Directional mRNA libraries were generated using poly(A) enrichment and sequenced on the NovaSeq X Plus platform (Illumina), generating paired‐end 150 bp reads. Library preparation and sequencing were performed by Novogene USA.

Paired‐end libraries from primary dermal fibroblasts and induced neurons (iNs) were processed independently with the nf‐core/rnaseq pipeline executed on Amazon Web Services EC2 under Nextflow v25.10.4, using Docker containerization for software reproducibility (Ewels et al. 2020). Fibroblast libraries were processed with nf‐core/rnaseq v3.23.0 against the iGenomes NCBI build of the human reference genome GRCh38, including the pre‐built STAR index and matching NCBI GTF annotation (Dobin et al. 2013). iN libraries were processed with nf‐core/rnaseq v3.20.0 against the Ensembl GRCh38 primary assembly with the Ensembl release 113 GTF. For both datasets, reads were adapter‐ and quality‐trimmed with Trim Galore (v0.6.10), aligned to the genome with STAR (v2.6.1d for fibroblasts and v2.7.11b for iNs), and transcript‐level abundances were quantified from the genome alignments with Salmon (v1.10.3) in alignment‐based mode. Gene‐level count matrices were generated by the pipeline's built‐in tximeta/tximport aggregation step (salmon.merged.gene_counts.tsv). Per‐sample sequencing and alignment quality were assessed with FastQC (v0.12.1), Picard MarkDuplicates, RSeQC, Qualimap, and dupRadar, and aggregated into MultiQC reports; all samples met quality thresholds (min_trimmed_reads = 10,000; min_mapped_reads = 5) prior to downstream statistical analysis.

All downstream analyses were performed in R. Gene‐level counts were imported into DESeq2, and genes with fewer than 10 total counts across all samples were removed prior to analysis. A multi‐factor differential expression model estimating age and sex effects simultaneously (design: ∼ sex + age) was fit. DESeq2 applied a negative binomial generalized linear model with empirical Bayes dispersion shrinkage and Wald test statistics. Log_2_ fold changes were further regularized using adaptive shrinkage (apeglm). Genes were considered significantly differentially expressed at an adjusted *p*‐value (Benjamini–Hochberg, BH) < 0.05 and |log_2_ fold change| > 1. For principal component analyses, variance‐stabilizing transformation (VST, blind = TRUE) was applied to the filtered count matrix and PCA was computed using prcomp(). Gene Ontology (GO) Biological Process enrichment was performed with enrichGO from clusterProfiler, using all tested genes as the background universe and converting HGNC gene symbols to Entrez IDs via org.Hs.eg.db; significance was defined at *p* < 0.05 and *q* < 0.05 (BH). Up‐ and downregulated gene sets were tested separately to preserve directional specificity. For gene set enrichment analysis (GSEA), all tested genes were pre‐ranked and analyzed using fgsea() with the c1.Hallmark, c2.KEGG:Medicus and c5.GO:BP gene sets. For hypothesis‐driven pathway analysis, curated gene sets were organized by PANTHER Biological Process and Cellular Component annotations, and selective autophagy receptor and CMA lists (Khawaja et al. 2025). VST expression values (blind = FALSE, using the ∼ sex + age design) were computed from the multi‐factor DESeq2 object and collapsed to the gene‐symbol level by averaging across multiple Ensembl IDs mapping to the same symbol. Gene expression was z‐scored row‐wise across samples, and a module score was computed per sample as the mean z‐score across all module genes. Module scores were compared between Young and Older groups using Welch's two‐sample *t*‐test (var.equal = FALSE). *p*‐values were corrected for multiple comparisons across all modules using the Benjamini–Hochberg method. Modules were considered nominally significant at *p* < 0.05; no modules reached FDR < 0.05, consistent with limited statistical power at *N* = 9.

### 4.7 Autophagy Flux Assay in Primary Dermal Fibroblasts

SHOCK primary dermal fibroblasts were seeded in fibroblast media in four 384‐well plates over a period of 2 days. On Day 1, 22 fibroblast cultures were seeded at a density of 150 cells per well, and on Day 2, the remaining 22 fibroblast cultures were seeded at a density of 250 cells per well. A 70% total volume media change was performed on all wells on Day 4. On Day 6, cells were subjected to an autophagy flux assay. For the autophagy flux assay, 50% of fibroblast media was removed from each well and replaced with either plain fibroblast media or fibroblast media containing 100 μM chloroquine (CQ; Sigma #C6628) for a final concentration of 50 μM CQ per well. Cells were incubated for 2 h at 37°C, 5% CO_2_. Media was then replaced with 4% paraformaldehyde (PFA) in DPBS for 20 min. Cells were then rinsed 3 times for 5 min with DPBS and processed for immunofluorescent staining by incubating in 1:100 anti‐LC3B (MBL Life Science #M152‐3) in 0.2% saponin (Acros Organics #419231000)/10% FBS in DPBS at 4°C for 1 h 30, followed by 1:1000 Alexa Fluor 488‐labeled secondary antibody (Invitrogen # A11001) and 1X Alexa Fluor 647‐labeled phalloidin (Thermo Scientific #A22287) in 0.2% saponin/10% FBS in DPBS at room temperature for 1 h, and finally 1X DAPI (Sigma #D9542) in DPBS for 15 min.

### 4.8 Autophagy Flux Assay in iNs

On day 23 post‐induction, iNs were separated by MACS as described above. Purified iNs were seeded in 12 Geltrex (Gibco #A1413201)‐coated wells of a 384‐well plate per cell line at 6000 cells per well in NK media. 24 h after seeding, iNs were transduced with pCDH‐EF1a‐mCherry‐EGFP‐LC3B (Addgene #170446) at MOI 20 in NK media containing 8 μg/mL polybrene. After 12 h transduction, wells were cycled with fresh NK media 5 times to remove the lentivirus. 72 h after lentivirus removal, an autophagy flux assay was performed as described above. Briefly, iNs were incubated in NK media at a final concentration of 0 or 50 μM CQ for 2 h at 37°C, 5% CO_2_. After treatment, media was replaced with ice cold PFA at a final concentration of 4% in DPBS for 20 min. Cells were then rinsed 3 times for 5 min with DPBS. iNs were processed for immunofluorescent staining by incubating in 1:500 anti‐TUJ1 (neuronal marker, BioLegend #801202) in 0.2% saponin/10% FBS in DPBS at 4°C overnight, followed by 1:1000 Alexa Fluor 647‐labeled secondary antibody (Invitrogen #A32728) in 0.2% saponin/10% FBS in DPBS at room temperature for 4 h, and finally, 1X DAPI in DPBS for 15 min.

### 4.9 Image Acquisition and Quantification of LC3B‐Positive Structures

Cells were imaged on an Opera Phenix High‐Content Screening system in confocal mode with a 40X objective. In primary dermal fibroblasts, total cell and LC3B‐positive foci area was quantified using a custom CellProfiler v4.2.8 (Stirling et al. 2021) pipeline in which LC3B‐positive foci were identified based on adaptive intensity and masked by phalloidin staining to assign to the correct cell. Foci were quantified in at least 81 fields of view per condition. Autophagy flux was determined by subtracting the average total LC3B‐positive foci area per cell area in CTRL‐treated cells from that in CQ‐treated cells. In iNs, total cell body and GFP‐LC3B‐positive foci area in TUJ1‐positive iNs was quantified by manually outlining and measuring the area of regions of interest using ImageJ2 v2.16.0/1.54p. Foci were quantified in at least 100 cells per condition. Autophagy flux was determined by subtracting the average total GFP‐LC3B‐positive foci area per cell body area in CTRL‐treated cells from that in CQ‐treated cells.

### 4.10 Autophagy Flux Assay and Isolation of Fresh Human PBMCs

Autophagy flux assays in PBMCs were performed similarly to a previously described method (Bensalem et al. 2021). Blood was drawn by a licensed phlebotomist into a 9 mL VACUETTE lithium heparin collection tube (Greiner #455084). Samples were processed within 45 min for SHOCK cohort participants, and within 2.5 h for STRONG cohort participants. After an initial Coulter count (Beckman Coulter DxH 520 hematology analyzer), blood was split evenly into three 9 mL VACUETTE lithium heparin tubes. Prostaglandin E (50 μL in DPBS; 18 μM stock) and CQ (final concentrations 0, 75 or 200 μM) were added to each sample. Samples were incubated at 37°C on a rotator (10 rpm) for 1 h prior to PBMC isolation. For PBMC isolation, samples were centrifuged at 500 x g for 15 min to separate the plasma from hematocrit. The hematocrit containing PBMCs was diluted in 12 mL warm Roswell Park Memorial Institute Medium (RPMI, Gibco #11‐835‐030) containing 50 μL prostaglandin E, layered over 3 mL Histopaque 1077 (Sigma #10771), and centrifuged at 700 × *g* for 30 min. The buffy coat was collected, diluted in 20 mL RPMI + 50 μL prostaglandin E, and centrifuged at 700 × *g* for 15 min. The PBMC pellet was resuspended in 950 μL PRMI + 50 μL prostaglandin E for a final Coulter count, then pelleted at 500 × *g* for 5 min and lysed in ice‐cold lysis buffer (10 mM Tris/HCl, pH 7.0, 1 mM EDTA, 0.5 mM EGTA, 1% Triton X‐100, 0.1% sodium deoxycholate, 0.1% SDS, 140 mM NaCl, 2.5 mM sodium pyrophosphate, 1 mM sodium orthovanadate, 1 mM 2‐glycerophosphate, 1X cOmplete protease inhibitor, 1X PhosSTOP phosphatase inhibitor in water). Lysates were incubated at 4°C on a rotator for 45–60 min, centrifuged at 16,000 × *g* at 4°C for 10 min, and the supernatant was aliquoted and flash‐frozen in liquid N_2_, and stored at −80°C. At a later timepoint, total protein concentration was determined using Pierce 660 Protein Assay Reagent (Thermo #22660) with 1X Ionic Detergent Compatibility Reagent (Thermo #22663).

### 4.11 Western Blots

For Western blotting, 1.5 μg of protein was mixed with 6X Laemmli buffer (Thermo #J61337.AD) and brought to a final volume of 36 μL with lysis buffer. Protein was denatured at 95°C for 10 min, after which samples were loaded onto a 10% Bis‐Tris SDS‐PAGE gel (NuPAGE #NP0315) in MOPS running buffer (NuPAGE, #NP0001), and separated at 110 V. Proteins were transferred to a PVDF membrane (Bio‐Rad Laboratories, #1704156) using the Trans‐Blot Turbo system (Bio‐Rad Laboratories) at 1.3 A for 7 min. The membrane was cut horizontally at ~30 kDa. The bottom portion was probed with rabbit anti‐LC3B (1:1000 in 1% milk in TBS‐T; Cell Signaling #3868) and secondary anti‐rabbit IgG HRP (1:10,000 in 1% milk in TBS‐T; Cell Signaling #7074), and the top portion was probed with mouse anti‐tubulin (1:1000 in 1% milk in TBS‐T; Cell Signaling #3873) and secondary anti‐mouse IgG HRP (1:10,000 in 1% milk in TBS‐T; Cell Signaling #7076). Blots were developed using SuperSignal chemiluminescence reagent (Thermo Scientific, #34577 or #34095) and imaged on a ChemiDoc imager (Bio‐Rad Laboratories Inc.). Protein expression was quantified using ImageJ2 (v2.16.0/1.54p) by measuring mean gray values within equal‐sized rectangular regions of interest. Background signal was calculated from three evenly spaced regions and subtracted from each measurement. LC3B‐II signal was normalized to tubulin, and autophagy flux was calculated as the difference between tubulin‐normalized LC3B‐II signal in CQ‐treated and CTRL‐treated samples.

### 4.12 Quantification and Statistical Analysis

Statistics for all RNA‐seq analyses were calculated using R as described above. All other statistics including Spearman correlation analyses, simple and multiple linear regression models, *t*‐tests, and graph generation were performed using Prism v10.6.0 (GraphPad). Statistical tests and significance are specified in corresponding figure legends.

## Author Contributions

**Tatiana M. Moreno:** conceptualization, data curation, formal analysis, funding acquisition, investigation, methodology, resources, software, supervision, validation, visualization, writing – original draft, writing – review and editing. **Stephanie R. Heimler:** data curation, investigation, methodology, resources, validation. **Ryan J. Moran:** conceptualization, funding acquisition, project supervision, writing – review. **Hava Shoshana Barkai:** data curation, investigation, writing – review. **Lina Scandalis:** data curation, project administration, resources. **Larissa Traxler:** data curation, investigation, methodology, resources, writing – review and editing. **Ashley Neil:** data curation, investigation, resources, writing – review and editing. **Stephen Dozier:** methodology, resources. **Jaclyn Bergstrom:** formal analysis, writing – review and editing. **Alexandre R. Colas:** conceptualization. **Sanjeev S. Ranade:** data curation, formal analysis, resources, software, writing – review and editing. **Anne G. Bang:** conceptualization, resources. **Jerome Mertens:** conceptualization, resources. **David Wing:** conceptualization, investigation, methodology, project supervision, writing – review. **Anthony J. Molina:** conceptualization, resources. **Caroline Kumsta:** conceptualization, funding acquisition, project administration, resources, supervision, writing – review and editing.

## Funding

This work was supported by the National Institute on Aging (R01‐AG083373 (to C.K.), R01‐AG085634 (to J.M.), U19‐AG023122 (to J.M.), P30‐AG068635 (San Diego Nathan Shock Center), P01‐AG073084‐04 (to Razavi Newman Integrative Genomics and Bioinformatics Core of the Salk Intitute)), American Foundation for Aging Research (AFAR‐30376715 (to J.M.)), Conrad Prebys Foundation (Predoctoral fellowship to T.M.M.), National Cancer Institute (P30‐CA030199, S10‐OD036254, P30‐CA014195). The Howard and Maryam Newman Family Foundation, and Leona M. and Harry B. Helmsley Charitable Trust.

## Conflicts of Interest

The authors declare no conflicts of interest.

## Supporting information


**Figure S1:** Global and autophagy‐related differential gene expression in young and older primary dermal fibroblasts and iNs. (A) Volcano plot of differentially‐expressed genes in older versus young primary dermal fibroblasts (|log_2_ fold change cutoff| ≥ 1.0; *p*_adj_ < 0.05). (B) *Z*‐scored expression of GO Biological Process macroautophagy genes in primary dermal fibroblasts ordered by ascending age. (C) PCA of primary dermal fibroblast and iN samples labeled by cell type and donor sex. (D) Volcano plot of differentially‐expressed genes in older versus young iNs (|log_2_ fold change cutoff| ≥ 1.0; *p*_adj_ < 0.05). (E) *Z*‐scored expression of GO Biological Process macroautophagy genes in iNs ordered by ascending age. *Statistical analysis*: Differential expression in (A) and (D) was determined using DESeq2 with Benjamini‐Hochberg adjustment (*p*_adj_ < 0.05). Each sample represents one biological replicate (independent donor; fibroblasts: *N* = 4 young, *N* = 5 older; iNs: *N* = 8 young, *N* = 5 older).
**Figure S2:** Chloroquine treatment reveals autophagy flux in primary dermal fibroblasts and iNs. (A) Spearman correlation coefficients (left) and corresponding *p*‐values (right) of autophagy flux assay measurements from assays performed in primary dermal fibroblasts from SHOCK cohort donors (*N* = 44). (B) Correlation between autophagy flux and mean LC3B‐positive puncta area per cell area following 50 μM CQ treatment in primary dermal fibroblasts, with each point representing one donor. Linear regression shown (*R* ^2^ and *p*‐values from linear regression model). (C) Spearman correlation coefficients (left) and corresponding *p*‐values (right) of autophagy flux assay measurements from assays performed in iNs from SHOCK cohort donors (*N* = 22). (D) Correlation between autophagy flux and mean LC3B‐positive puncta area per cell area at 50 μM CQ treatment in iNs, with each point representing one donor. Linear regression shown (*R* ^2^ and *p*‐values from linear regression model). Autophagy flux is defined as the CQ‐induced change in LC3B or GFP‐LC3B‐positive puncta area per cell (CQ‐CTRL).
**Figure S3:** LC3B‐positive foci area is not associated with cell size following chloroquine treatment in primary dermal fibroblasts and iNs.
(A) Mean LC3B‐positive foci area per cell area in primary dermal fibroblasts from male SHOCK cohort donors treated ± CQ (*N* = 23). Linear regression per treatment by age (*R* ^2^_CTRL_ = 0.03; *R* ^2^_CQ_ = 0.10); comparison of CTRL versus CQ slopes, *p* = 0.09; comparison of CTRL versus CQ intercepts, *p* < 0.0001. (B) Mean LC3B‐positive foci area per cell area in primary dermal fibroblasts from female SHOCK cohort donors treated ± CQ (*N* = 21). Linear regression per treatment by age (*R* ^2^_CTRL_ = 0.10; R^2^_CQ_ = 0.03); comparison of CTRL versus CQ slopes, *p* = 0.91; comparison of CTRL versus CQ intercepts, *p* < 0.0001. (C) Mean cell area of primary dermal fibroblasts from male SHOCK cohort donors treated ± CQ (*N* = 23). Comparison of CTRL versus CQ, *p* = 0.13 by Wilcoxon matched‐pairs signed rank test. (D) Mean cell area of primary dermal fibroblasts from female SHOCK cohort donors treated ± CQ (*N* = 21). Comparison of CTRL versus CQ, *p* > 0.99 by Wilcoxon matched‐pairs signed rank test. (E) Mean GFP‐LC3B‐positive foci area per cell body area in iNs from male SHOCK cohort donors treated ± CQ (*N* = 14). Linear regression per treatment by age (*R* ^2^_CTRL_ = 0.05; *R* ^2^_CQ_ = 0.01); comparison of CTRL versus CQ slopes, *p* = 0.52; comparison of CTRL versus CQ intercepts, *p* = 0.06. (F) Mean GFP‐LC3B‐positive foci area per cell body area in iNs from female SHOCK cohort donors treated ± CQ (*N* = 8). Linear regression per treatment by age (*R* ^2^_CTRL_ = 0.03; *R* ^2^_CQ_ = 0.38); comparison of CTRL versus CQ slopes, *p* = 0.09; comparison of CTRL versus CQ intercepts, *p* = 0.03. (G) Mean cell body area of iNs from male SHOCK cohort donors treated ± CQ (*N* = 14). Comparison of CTRL versus CQ, *p* = 0.09 by Wilcoxon matched‐pairs signed rank test. (H) Mean cell body area of iNs from female SHOCK cohort donors treated ± CQ (*N* = 8). Comparison of CTRL versus CQ, *p* > 0.74 by Wilcoxon matched‐pairs signed rank test.
**Figure S4:** PBMC isolation enriches for lymphocytes and yields consistent cell composition across samples. (A) PBMC composition in whole blood and isolated PBMC samples from SHOCK cohort participants, measured by Coulter counter analysis (*N* = 23). Mean ± standard deviation. Statistical comparison of cell type abundance before and after PBMC isolation were performed by two‐way ANOVA; ns—*p* > 0.05; *p < 0.05; **p < 0.01; ***p < 0.001, ****p < 0.0001. (B) Coefficient of variation (CV) of PBMC cell type abundance (% of total cells) across 0, 75 and 200 μM CQ‐treated samples from SHOCK participants. Each data point represents the CV calculated from 3 treatment conditions for one participant. Dotted line indicates CV threshold of 20%.
**Figure S5:** Chloroquine treatment of freshly drawn whole blood reveals autophagy flux in human PBMCs. (A) Spearman correlation coefficients (left) and corresponding *p*‐values (right) of autophagy flux assay measurements in freshly isolated PBMCs from SHOCK cohort donors (*N* = 23). (B) Correlation between autophagy flux and LC3B‐II levels following 200 μM CQ treatment, with *R* ^2^ and *p*‐values from linear regression analysis. The log_10_‐scaled *x*‐axis results in the curved appearance of the linear trend line.
**Figure S6:** PBMC composition in whole blood and isolated PBMCs does not correlate with age or autophagy flux. (A) Relationship between age and total white blood cell counts (WBC; top), and PBMC cell type composition (bottom) in whole blood from SHOCK cohort participants. Each data point represents one participant. *R* ^2^ values and p_slope_ derived from linear regression. (B) Relationship between total white blood cell counts (WBC; top), and PBMC cell type composition (bottom) with age (left) and autophagy flux (right) in SHOCK cohort participant isolated PBMC samples. Each data point represents the mean abundance of the indicated cell type across 0, 75 and 200 μM CQ‐treated samples for each individual.
**Figure S7:** Changes in SHOCK cohort health and fitness with age. Health and fitness readouts for all SHOCK participants (*N* = 49–58) as a function of age. (A) resting VO_2_; (B) maximum heart rate; (C) VO_2max_; (D) maximum minute ventilation (VE); (E) maximal grip strength of strongest hand; (F) average very vigorous minutes per day; (G) walking speed in a 6‐min walking test; (H) systolic blood pressure; (I) diastolic blood pressure; (J) SPPB test chair stand time; (K) body mass index (BMI); (L) resting heart rate; (M) short physical performance battery (SPPB) score out of 12; (N) average sedentary minutes per day.
**Figure S8:** Comparison of health and fitness readouts in STRONG participants between Week 0 and Week 12 of mild exercise. Week 0 and Week 12 STRONG participant (*N* = 5) (A) body mass index (BMI); (B) resting heart rate; (C) maximal grip strength of strongest hand; (D) occiput to wall distance standing with a normal posture; (E) occiput to wall distance standing as tall as possible; (F) systolic blood pressure; (G) diastolic blood pressure; (H) SPPB test chair stand time. *p*‐values by paired *t*‐tests.
**Figure S9:** Comparison of health and fitness readouts in SHOCK PBMC donors and STRONG participants. (A) SHOCK and STRONG participant age. SHOCK PBMC donor (*N* = 23), and week 0 STRONG participant (*N* = 5) (B) body mass index (BMI); (C) short physical performance battery (SPPB) score; (D) maximal grip strength of strongest hand; (E) resting heart rate; (F) systolic blood pressure; (G) diastolic blood pressure; (H) SPPB test chair stand time. *p*‐values by Welch's *t*‐test.
**Figure S10:** PBMC composition in whole blood and isolated PBMCs is consistent in STRONG participant samples from Week 0 to Week 12. (A) PBMC composition in whole blood and isolated PBMC samples from SHOCK cohort participants on Week 0 and Week 12 of the exercise intervention, measured by Coulter counter analysis (*N* = 5). Mean ± standard deviation. Statistical comparison of cell type abundance between Week 0 and Week 12 of exercise within whole blood or isolated PBMC samples performed by two‐way ANOVA; ns—*p* > 0.05. (B‐C) Percent composition of STRONG participant PBMC subtypes in (B) whole blood and (C) isolated PBMC samples on Week 0 and Week 12 of exercise. Each data point represents the mean abundance of the indicated cell type in 0 and 200 μM CQ‐treated samples for each individual. *p*‐values by paired *t*‐tests.

**Table S1:** Experimental details, demographics and health measures of participants of the San Diego Nathan Shock Center Healthy Aging (“SHOCK”) cohort.
**Table S2:** PANTHER Slim‐GO Biological Process module scores in SHOCK fibroblasts from older versus younger donors.
**Table S3:** Significantly up‐ and downregulated GSEA terms with age in SHOCK primary dermal fibroblasts.
**Table S4:** Significantly up‐ and downregulated GSEA terms with age in SHOCK induced neurons.
**Table S5:** Summary of autophagy‐related gene modules defined by the PANTHER Classification System.
**Table S6:** Assignment of autophagy‐related genes to GO biological Process modules and regulatory categories.
**Table S7:** Demographics and health measures of the STRONG cohort before and after a 12‐week exercise intervention.
**Table S8:** Demographics and health measures of the SHOCK PBMC cohort and STRONG cohort before and after a 12‐week exercise intervention.

## Data Availability

Fibroblast and iN RNA‐seq data have been deposited at GEO under accession number GSE331495 and are publicly available as of the date of publication. Additional data that support the findings of this study are available from the corresponding author upon reasonable request.
