# Supplementary material for: Autophagy Flux Is Remodeled Sex‐ and Cell Type‐Specifically During Human Aging, and Is Linked to Reduced Physical Function in Older Adults
Source: Aging Cell. 2026 Sep 27;25(10):e70734. doi: 10.1111/acel.70734 (PMC13617198; doi:10.1111/acel.70734)
Supplement: Supplementary file 1 [file ACEL-25-e70734-s002.pdf]

Supplemental Information

Autophagy flux is remodeled sex- and cell type-specifically during human aging, and is associated with physical fitness

Tatiana M. Moreno, Stephanie R. Heimler, Ryan J. Moran, Hava Shoshana Barkai, Lina Scandalis, Larissa Traxler, Ashley Neil, Stephen Dozier, Jaclyn Bergstrom, Alexandre R. Colas, Sanjeev S. Ranade, Anne G. Bang, Jerome Mertens, David Wing, Anthony J. Molina, Caroline Kumsta

Supplemental document S1.

Contents

Figure S1. Global and autophagy-related differential gene expression in young and older primary dermal fibroblasts and iNs.

Figure S2. Chloroquine treatment reveals autophagy flux in primary dermal fibroblasts and iNs.

Figure S3. LC3B-positive puncta area is not associated with cell size following chloroquine treatment in primary dermal fibroblasts and iNs.

Figure S4. PBMC isolation enriches for lymphocytes and yields consistent cell composition across samples.

Figure S5. Chloroquine treatment of freshly drawn whole blood reveals autophagy flux in human PBMCs.

Figure S6. PBMC composition in whole blood and isolated PBMCs does not correlate with age or autophagy flux.

Figure S7. Changes in SHOCK cohort health and fitness with age.

Figure S8. Comparison of health and fitness readouts in STRONG participants between week 0 and week 12 of mild exercise.

Figure S9. Comparison of health and fitness readouts in SHOCK PBMC donors and STRONG participants.

Figure S10. PBMC composition in whole blood and isolated PBMCs is consistent in STRONG participant samples from week 0 to week 12.

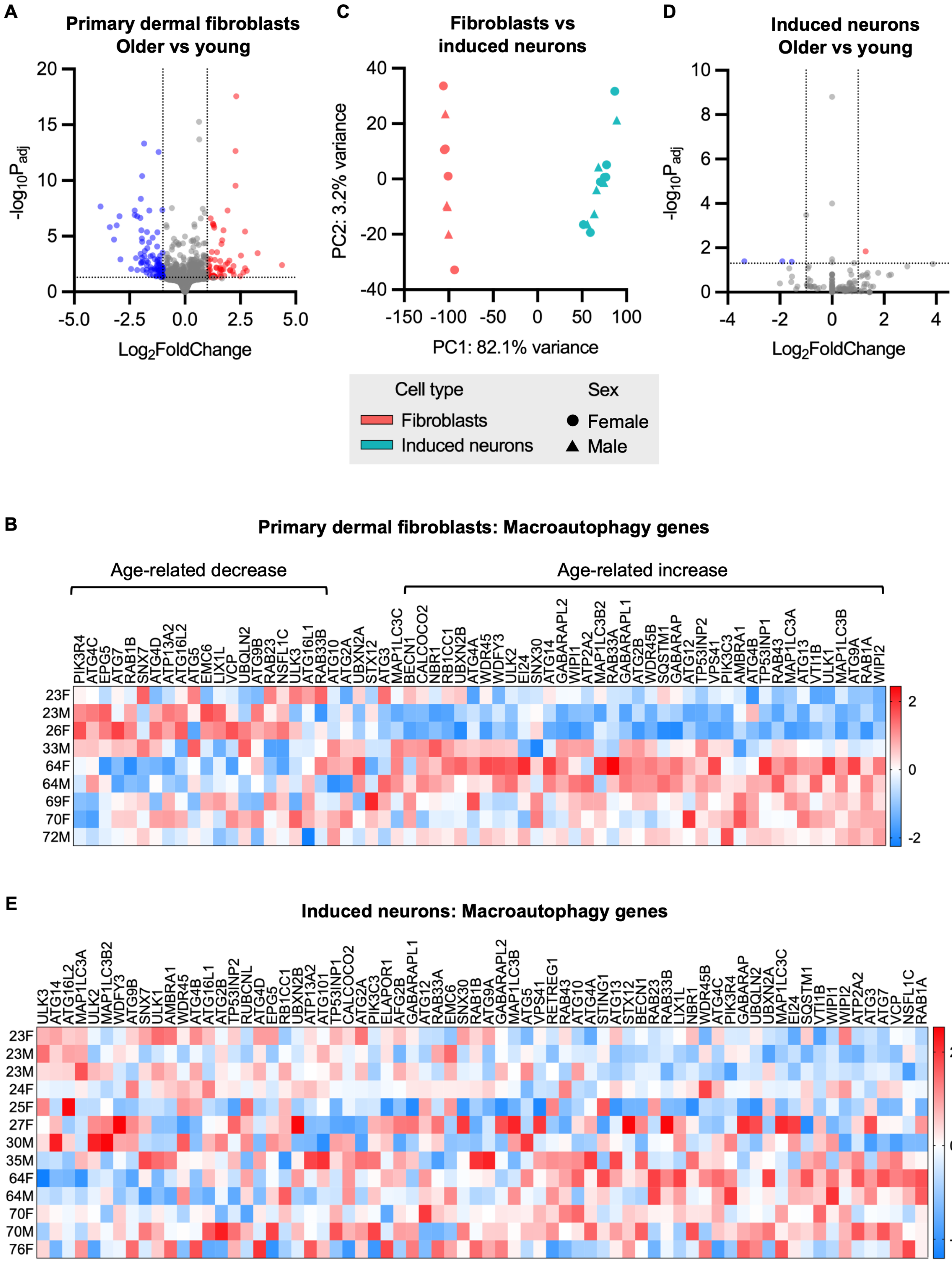
A
Primary dermal fibroblasts
Older vs young
$-\log_{10}P_{adj}$
Log$_2$FoldChange
20
15
10
5
0
-5.0
-2.5
0.0
2.5
5.0
C
Fibroblasts vs
induced neurons
PC2: 3.2% variance
PC1: 82.1% variance
40
20
0
-20
-40
-150
-100
-50
0
50
100
D
Induced neurons
Older vs young
$-\log_{10}P_{adj}$
Log$_2$FoldChange
10
8
6
4
2
0
-4
-2
0
2
4
Cell type
Fibroblasts
Induced neurons
Sex
Female
Male
B
Primary dermal fibroblasts: Macroautophagy genes
Age-related decrease
Age-related increase
PIK3R4 ATG4C EPG5 ATG7 RAB1B SNX7 ATG4D ATP13A2 ATG16L2 ATG5 EMC6 LIX1L VCP UBQLN2 ATG9B RAB23 NSFL1C ULK3 ATG16L1 RAB33B ATG10 ATG2A UBXN2A STX12 ATG3 MAP1LC3C BECN1 CALCOCO2 NBR1 RB1CC1 UBXN2B ATG4A WDR45 WDFY3 ULK2 EI24 SNX30 ATG14 GABARAPL2 WIPI1 ATP2A2 MAP1LC3B2 RAB33A GABARAPL1 ATG2B WDR45B SQSTM1 GABARAP ATG12 TP53INP2 VPS41 PIK3C3 AMBRA1 ATG4B TP53INP1 RAB43 MAP1LC3A ATG13 VTI1B ULK1 MAP1LC3B ATG9A RAB1A WIPI2
23F
23M
26F
33M
64F
64M
69F
70F
72M
2
0
-2
E
Induced neurons: Macroautophagy genes
ULK3 ATG14 ATG16L2 MAP1LC3A ULK2 MAP1LC3B2 WDFY3 ATG9B SNX7 ULK1 AMBRA1 WDR45 ATG4B ATG16L1 ATG2B TP53INP2 RUBCNL ATG4D EPG5 RB1CC1 UBXN2B ATP13A2 ATG101 TP53INP1 CALCOCO2 ATG2A PIK3C3 ELAPOR1 AFG2B GABARAPL1 ATG12 RAB33A EMC6 SNX30 RAB1B ATG9A GABARAPL2 MAP1LC3B ATG5 VPS41 RETREG1 RAB43 ATG10 ATG4A STING1 ATG13 STX12 BECN1 RAB23 RAB33B LIX1L NBR1 WDR45B ATG4C PIK3R4 GABARAP UBQLN2 UBXN2A MAP1LC3C EI24 SQSTM1 VTI1B WIPI1 WIPI2 ATP2A2 ATG3 ATG7 VCP NSFL1C RAB1A
23F
23M
23M
24F
25F
27F
30M
35M
64F
64M
70F
70M
76F
2
0
-2

Figure S1. Global and autophagy-related differential gene expression in young and older primary dermal fibroblasts and iNs.

(**A**) Volcano plot of differentially-expressed genes in older vs young primary dermal fibroblasts (|$\log_2$ fold change cutoff| ≥ 1.0; $p_{adj} < 0.05$). (**B**) Z-scored expression of GO Biological Process macroautophagy genes in primary dermal fibroblasts ordered by ascending age. (**C**) PCA of primary dermal fibroblast and iN samples labeled by cell type and donor sex. (**D**) Volcano plot of differentially-expressed genes in older vs young iNs (|$\log_2$ fold change cutoff| ≥ 1.0; $p_{adj} < 0.05$). (**E**) Z-scored expression of GO Biological Process macroautophagy genes in iNs ordered by ascending age. Statistical analysis: Differential expression in (**A**) and (**D**) was determined using DESeq2 with Benjamini-Hochberg adjustment ($p_{adj} < 0.05$). Each sample represents one biological replicate (independent donor; fibroblasts: N = 4 young, N = 5 older; iNs: N = 8 young, N = 5 older).

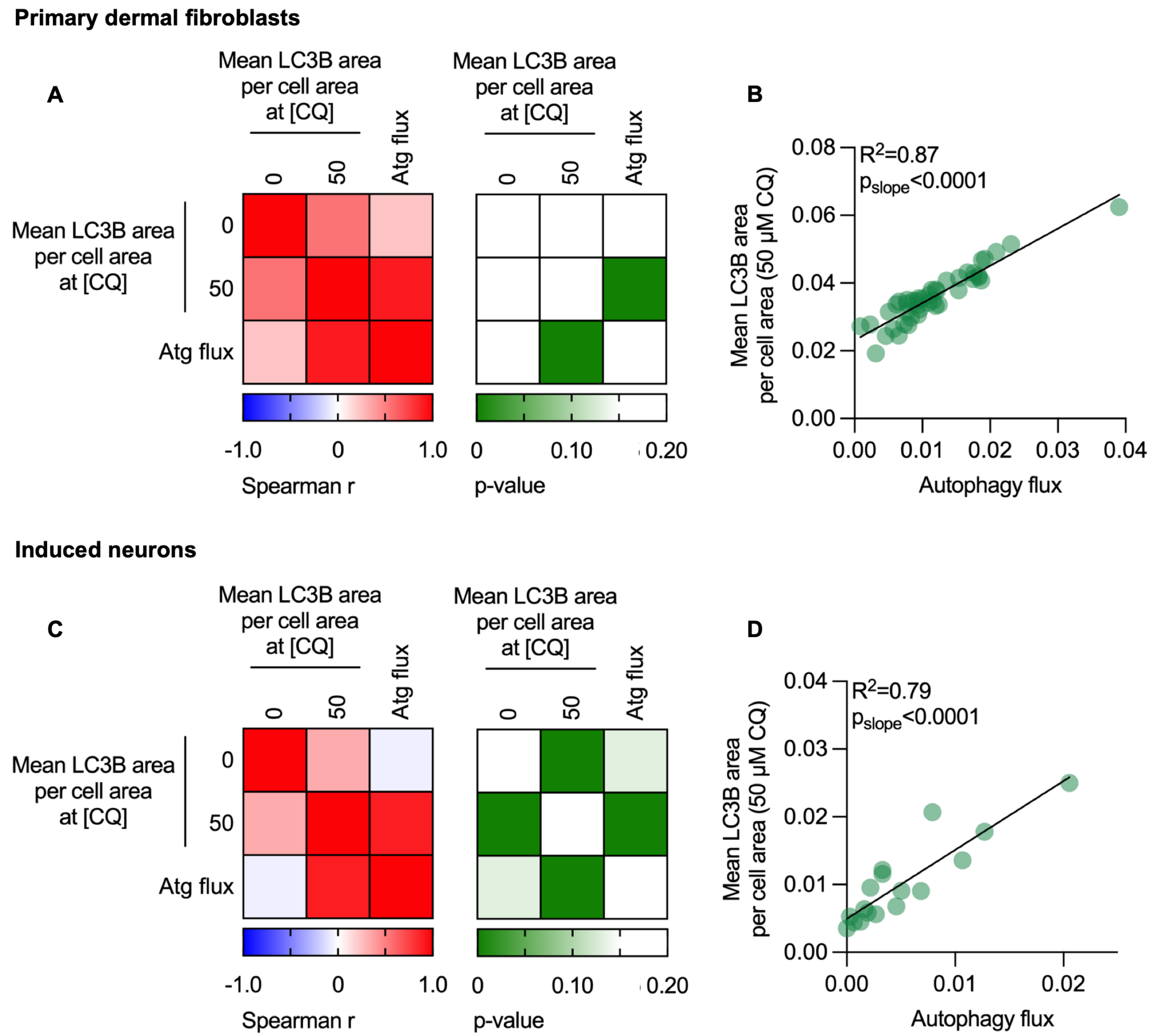

Figure S2. Chloroquine treatment reveals autophagy flux in primary dermal fibroblasts and iNs.
(**A**) Spearman correlation coefficients (left) and corresponding p-values (right) of autophagy flux assay measurements from assays performed in primary dermal fibroblasts from SHOCK cohort donors (N = 44). (**B**) Correlation between autophagy flux and mean LC3B-positive puncta area per cell area following 50 µM CQ treatment in primary dermal fibroblasts, with each point representing one donor. Linear regression shown (R^2 and p-values from linear regression model). (**C**) Spearman correlation coefficients (left) and corresponding p-values (right) of autophagy flux assay measurements from assays performed in iNs from SHOCK cohort donors (N = 17). (**D**) Correlation between autophagy flux and mean LC3B-positive puncta area per cell area at 50 µM CQ treatment in iNs, with each point representing one donor. Linear regression shown (R^2 and p-values from linear regression model). Autophagy flux is defined as the CQ-induced change in LC3B or GFP-LC3B-positive puncta area per cell (CQ-CTRL).

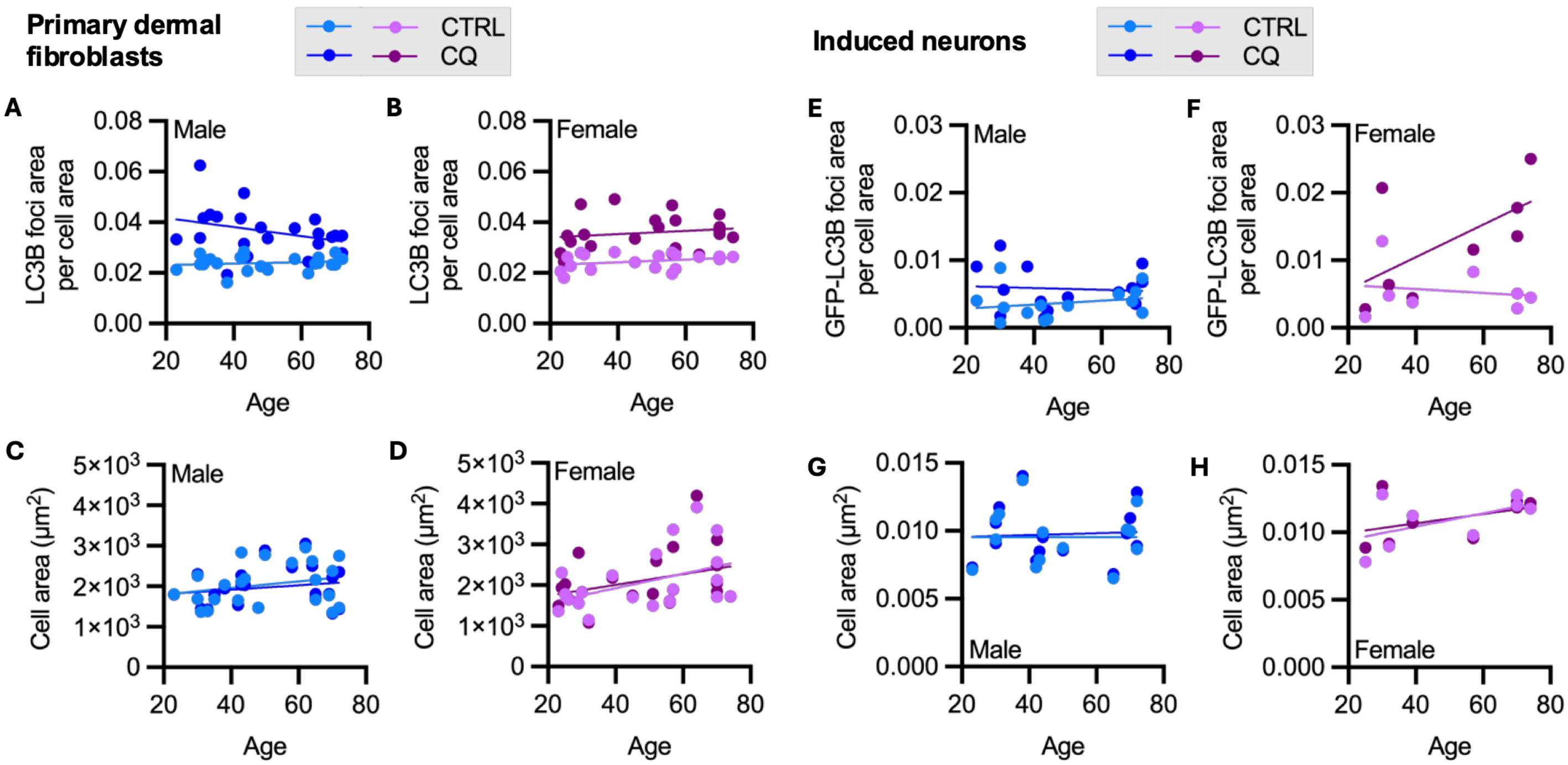

Figure S3. LC3B-positive foci area is not associated with cell size following chloroquine treatment in primary dermal fibroblasts and iNs.

(**A**) Mean LC3B-positive foci area per cell area in primary dermal fibroblasts from male SHOCK cohort donors treated ± CQ (N = 23). Linear regression per treatment by age (R^2_{CTRL}= 0.03; R^2_{CQ} = 0.10); comparison of CTRL vs CQ slopes, p = 0.09; comparison of CTRL vs CQ intercepts, p < 0.0001. (**B**) Mean LC3B-positive foci area per cell area in primary dermal fibroblasts from female SHOCK cohort donors treated ± CQ (N = 21). Linear regression per treatment by age (R^2_{CTRL} = 0.10; R^2_{CQ} = 0.03); comparison of CTRL vs CQ slopes, p = 0.91; comparison of CTRL vs CQ intercepts, p < 0.0001. (**C**) Mean cell area of primary dermal fibroblasts from male SHOCK cohort donors treated ± CQ (N = 23). Comparison of CTRL vs CQ, p = 0.13 by Wilcoxon matched-pairs signed rank test. (**D**) Mean cell area of primary dermal fibroblasts from female SHOCK cohort donors treated ± CQ (N = 21). Comparison of CTRL vs CQ, p > 0.99 by Wilcoxon matched-pairs signed rank test. (**E**) Mean GFP-LC3B-positive foci area per cell body area in iNs from male SHOCK cohort donors treated ± CQ (N = 14). Linear regression per treatment by age (R^2_{CTRL}= 0.05; R^2_{CQ} = 0.01); comparison of CTRL vs CQ slopes, p = 0.52; comparison of CTRL vs CQ intercepts, p = 0.06. (**F**) Mean GFP-LC3B-positive foci area per cell body area in iNs from female SHOCK cohort donors treated ± CQ (N = 8). Linear regression per treatment by age (R^2_{CTRL} = 0.03; R^2_{CQ} = 0.38); comparison of CTRL vs CQ slopes, p = 0.09; comparison of CTRL vs CQ intercepts, p = 0.03. (**G**) Mean cell body area of iNs from male SHOCK cohort donors treated ± CQ (N = 14). Comparison of CTRL vs CQ, p = 0.09 by Wilcoxon matched-pairs signed rank test. (**H**) Mean cell body area of iNs from female SHOCK cohort donors treated ± CQ (N = 8). Comparison of CTRL vs CQ, p > 0.74 by Wilcoxon matched-pairs signed rank test.

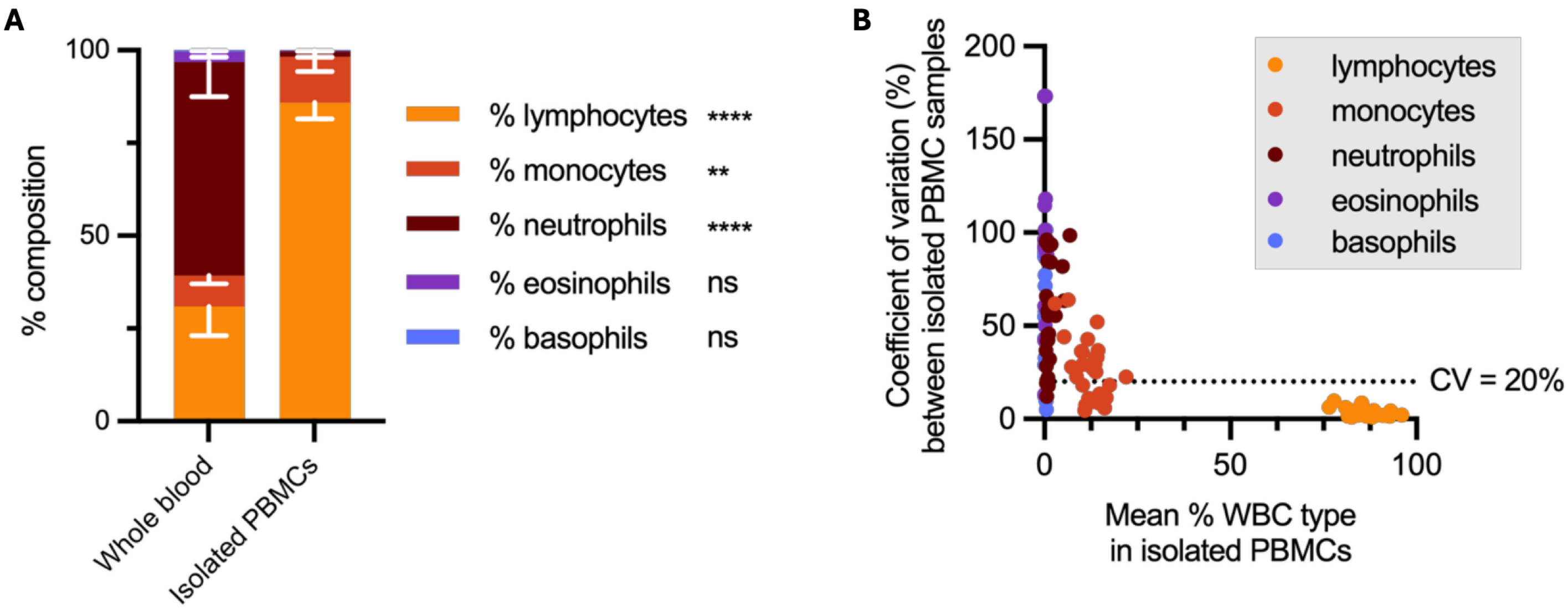

Figure S4. PBMC isolation enriches for lymphocytes and yields consistent cell composition across samples. (**A**) PBMC composition in whole blood and isolated PBMC samples from SHOCK cohort participants, measured by Coulter counter analysis (N = 23). Mean ± standard deviation. Statistical comparison of cell type abundance before and after PBMC isolation were performed by two-way ANOVA; ns – p>0.05; *p<0.05; **p<0.01; ***p<0.001, ****p<0.0001. (**B**) Coefficient of variation (CV) of PBMC cell type abundance (% of total cells) across 0, 75 and 200 μM CQ-treated samples from SHOCK participants. Each data point represents the CV calculated from 3 treatment conditions for one participant. Dotted line indicates CV threshold of 20%.

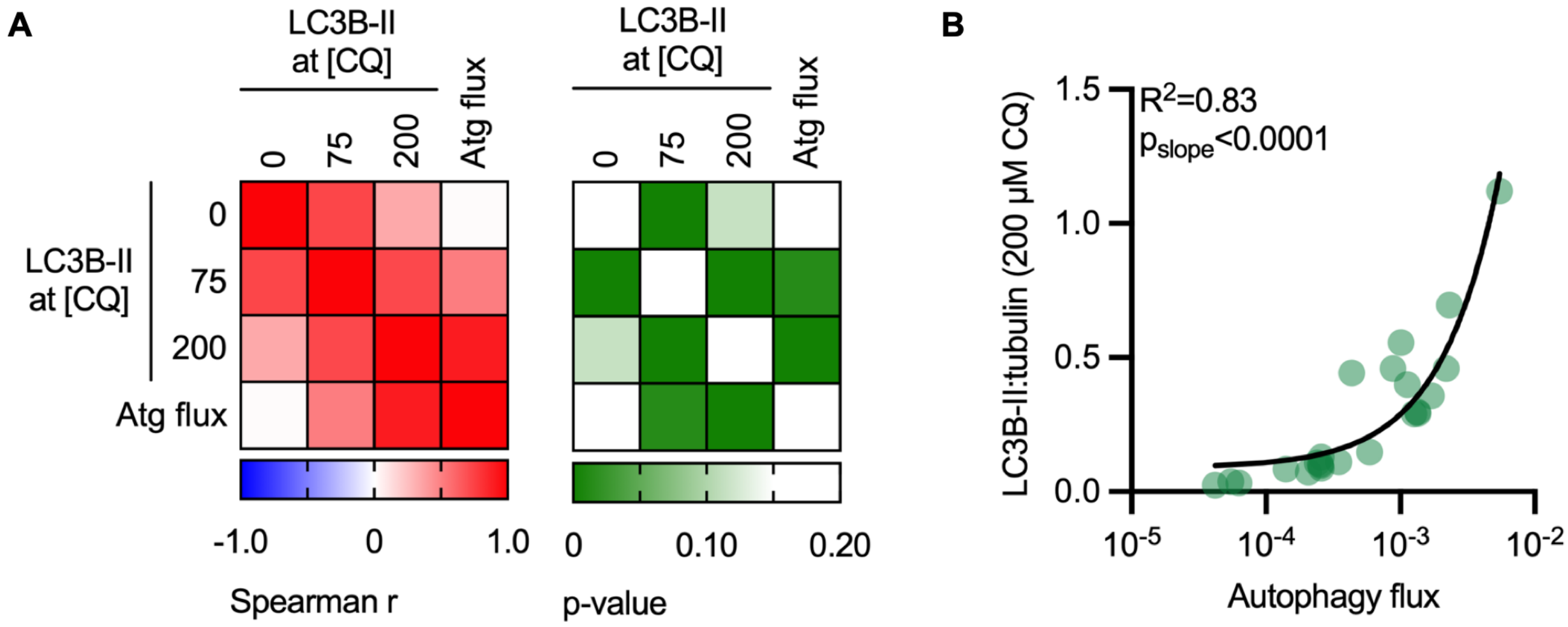

Figure S5. Chloroquine treatment of freshly drawn whole blood reveals autophagy flux in human PBMCs.
(**A**) Spearman correlation coefficients (left) and corresponding p-values (right) of autophagy flux assay measurements in freshly isolated PBMCs from SHOCK cohort donors (N = 23). (**B**) Correlation between autophagy flux and LC3B-II levels following 200 µM CQ treatment, with R^2 and p-values from linear regression analysis. The log_{10}-scaled x-axis results in the curved appearance of the linear trend line.

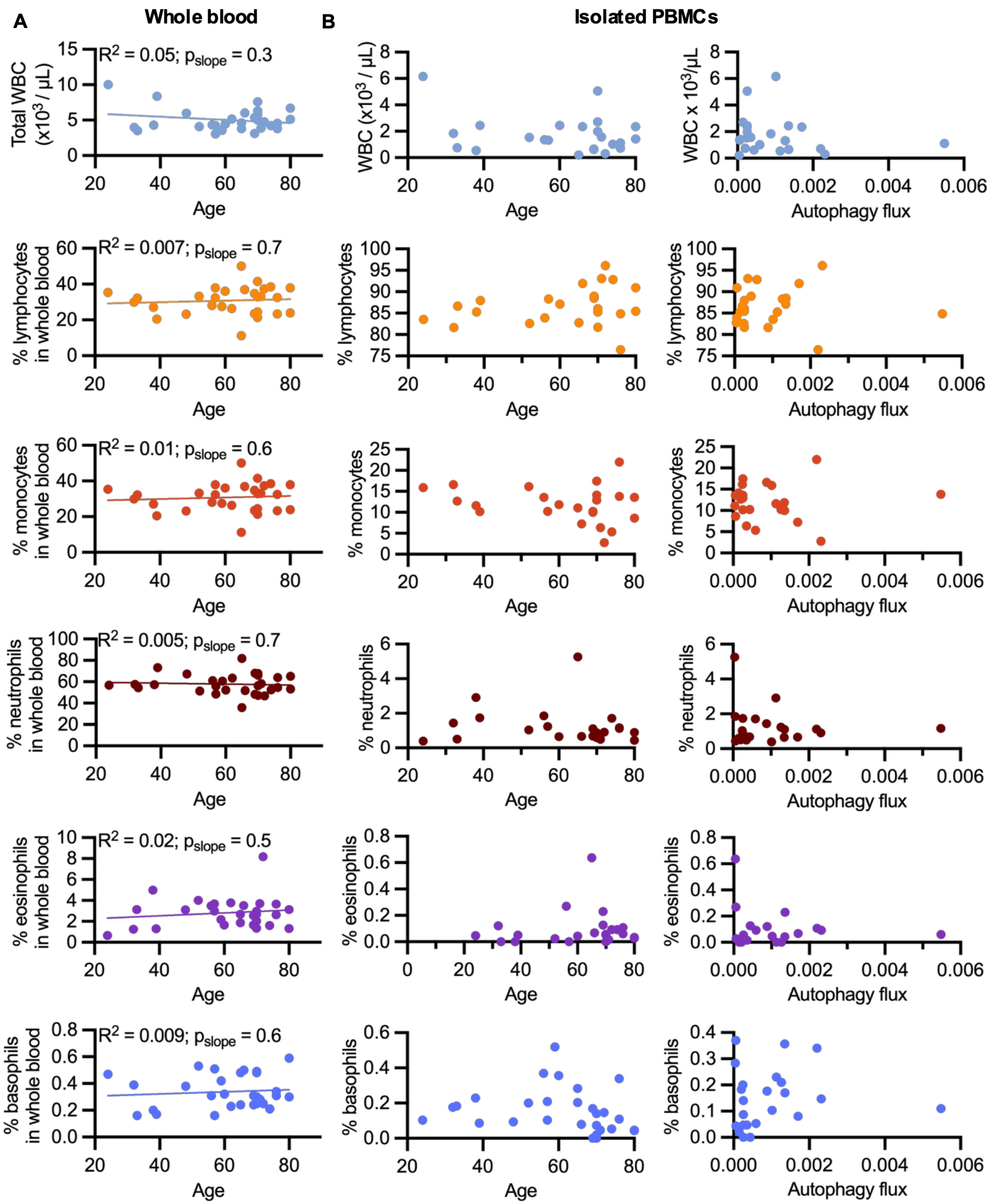

Figure S6. PBMC composition in whole blood and isolated PBMCs does not correlate with age or autophagy flux. (**A**) Relationship between age and total white blood cell counts (WBC; top), and PBMC cell type composition (bottom) in whole blood from SHOCK cohort participants. Each data point represents one participant. R^2 values and p_{slope} derived from linear regression. (**B**) Relationship between total white blood cell counts (WBC; top), and PBMC cell type composition

(bottom) with age (left) and autophagy flux (right) in SHOCK cohort participant isolated PBMC samples. Each data point represents the mean abundance of the indicated cell type across 0, 75 and 200 μM CQ-treated samples for each individual.

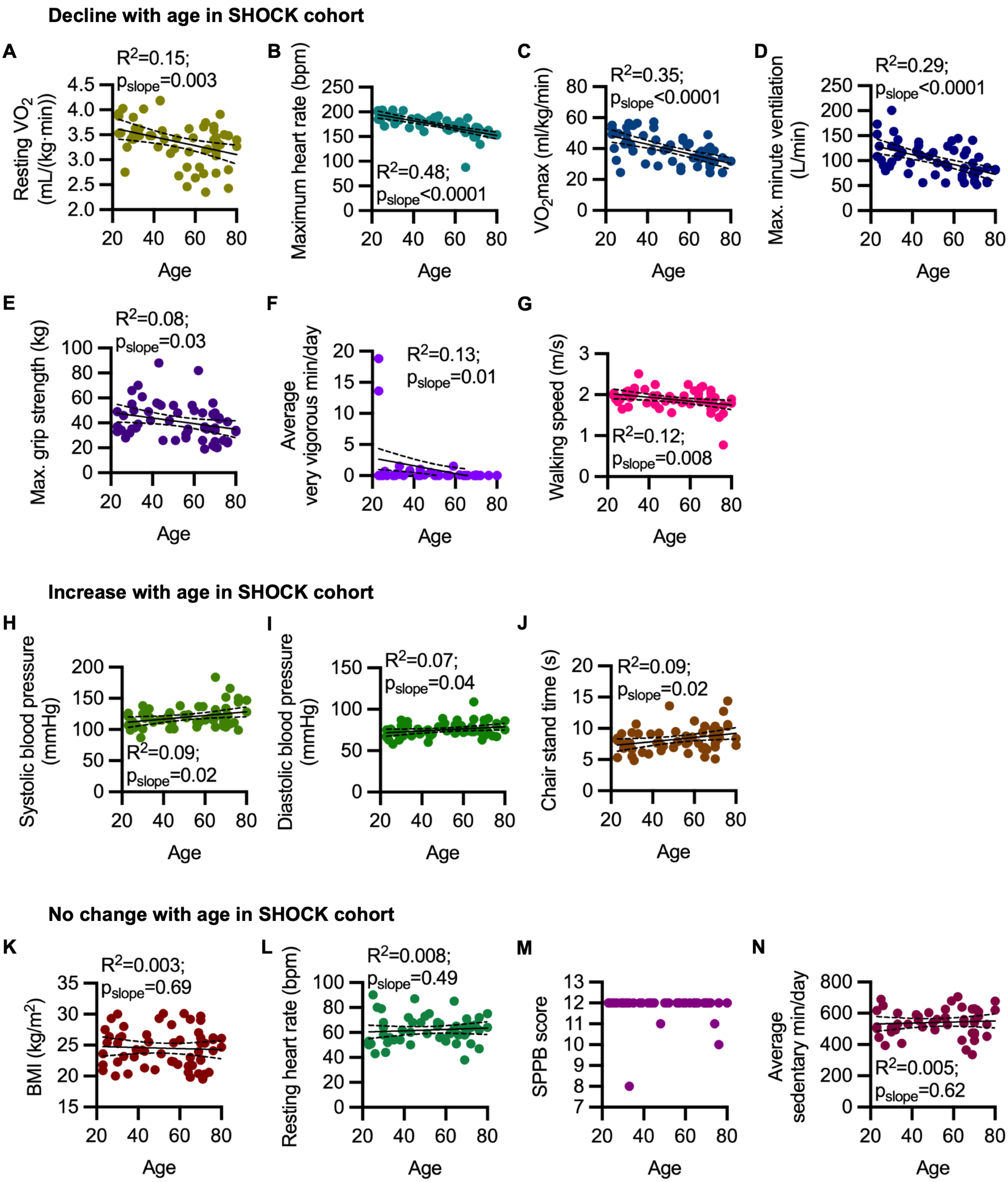

Figure S7. Changes in SHOCK cohort health and fitness with age.
Health and fitness readout for all SHOCK participants (N = 49-58) as a function of age. (**A**) resting VO_2; (**B**) maximum heart rate; (**C**) VO_{2max}; (**D**) maximum minute ventilation (VE); (**E**) maximal grip strength of strongest hand; (**F**) average very vigorous minutes per day; (**G**) walking speed in a 6-minute walking test; (**H**) systolic blood pressure; (**I**) diastolic blood pressure; (**J**) SPPB test chair stand time; (**K**) body mass index (BMI); (**L**) resting heart rate; (**M**) short physical performance battery (SPPB) score out of 12; (**N**) average sedentary minutes per day.

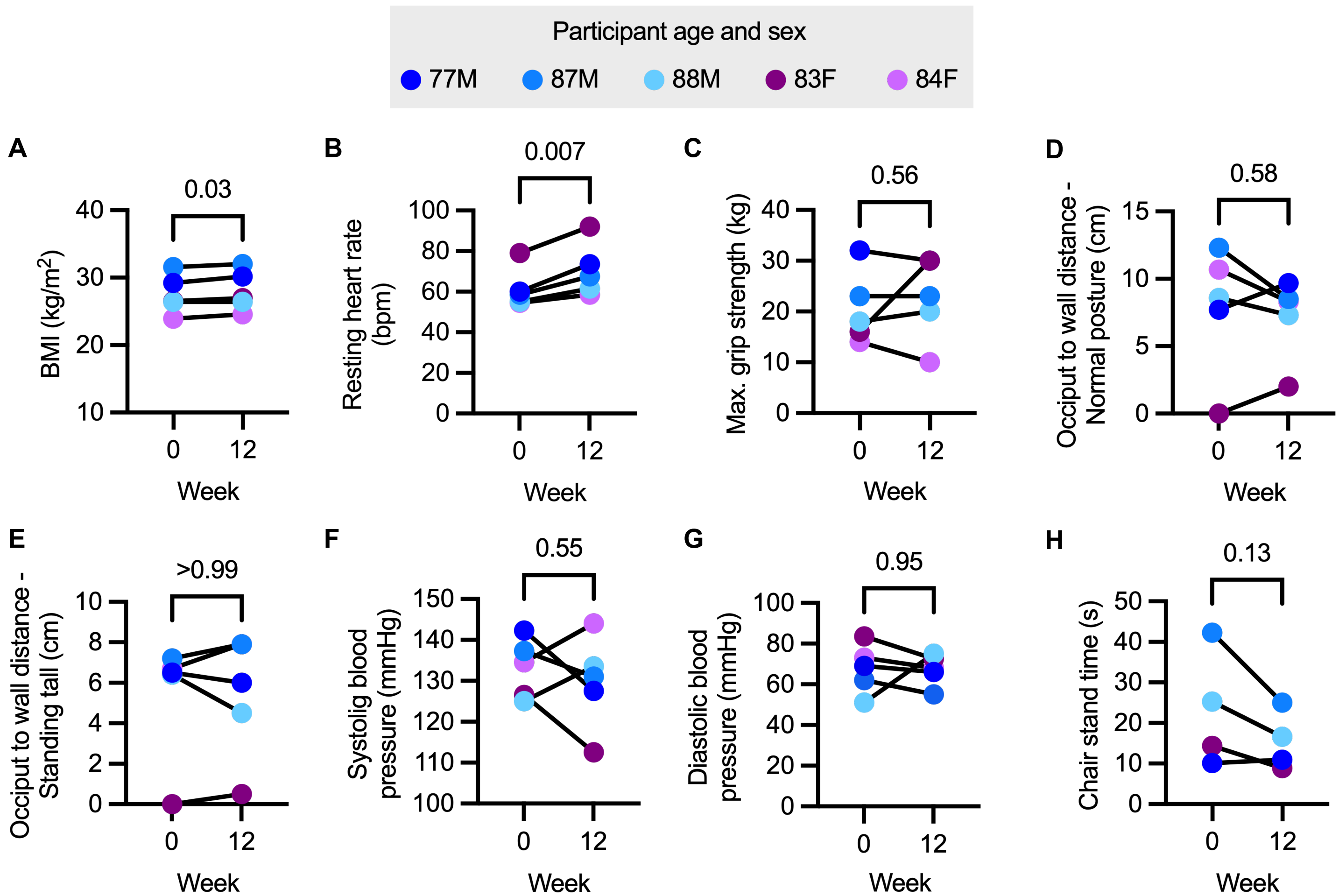

Figure S8. Comparison of health and fitness readouts in STRONG participants between week 0 and week 12 of mild exercise.

Week 0 and week 12 STRONG participant (N = 5) (**A**) body mass index (BMI); (**B**) resting heart rate; (**C**) maximal grip strength of strongest hand; (**D**) occiput to wall distance standing with a normal posture; (**E**) occiput to wall distance standing as tall as possible; (**F**) systolic blood pressure; (**G**) diastolic blood pressure; (**H**) SPPB test chair stand time. P-values by paired t-tests.

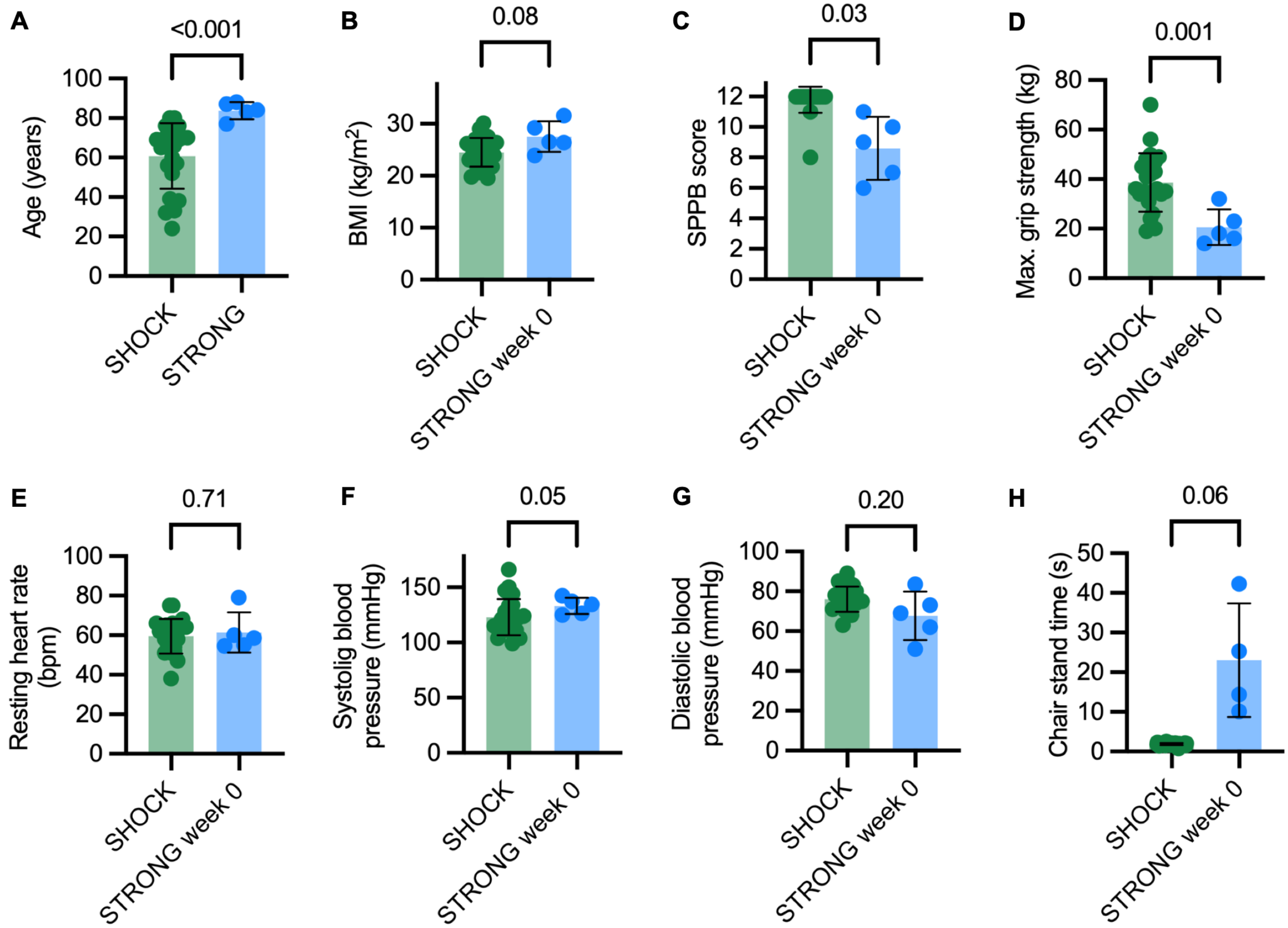

Figure S9. Comparison of health and fitness readouts in SHOCK PBMC donors and STRONG participants. (**A**) SHOCK and STRONG participant age. SHOCK PBMC donor (N = 23), and week 0 STRONG participant (N = 5) (**B**) body mass index (BMI); (**C**) short physical performance battery (SPPB) score; (**D**) maximal grip strength of strongest hand; (**E**) resting heart rate; (**F**) systolic blood pressure; (**G**) diastolic blood pressure; (**H**) SPPB test chair stand time. P-values by Welch's t-test.

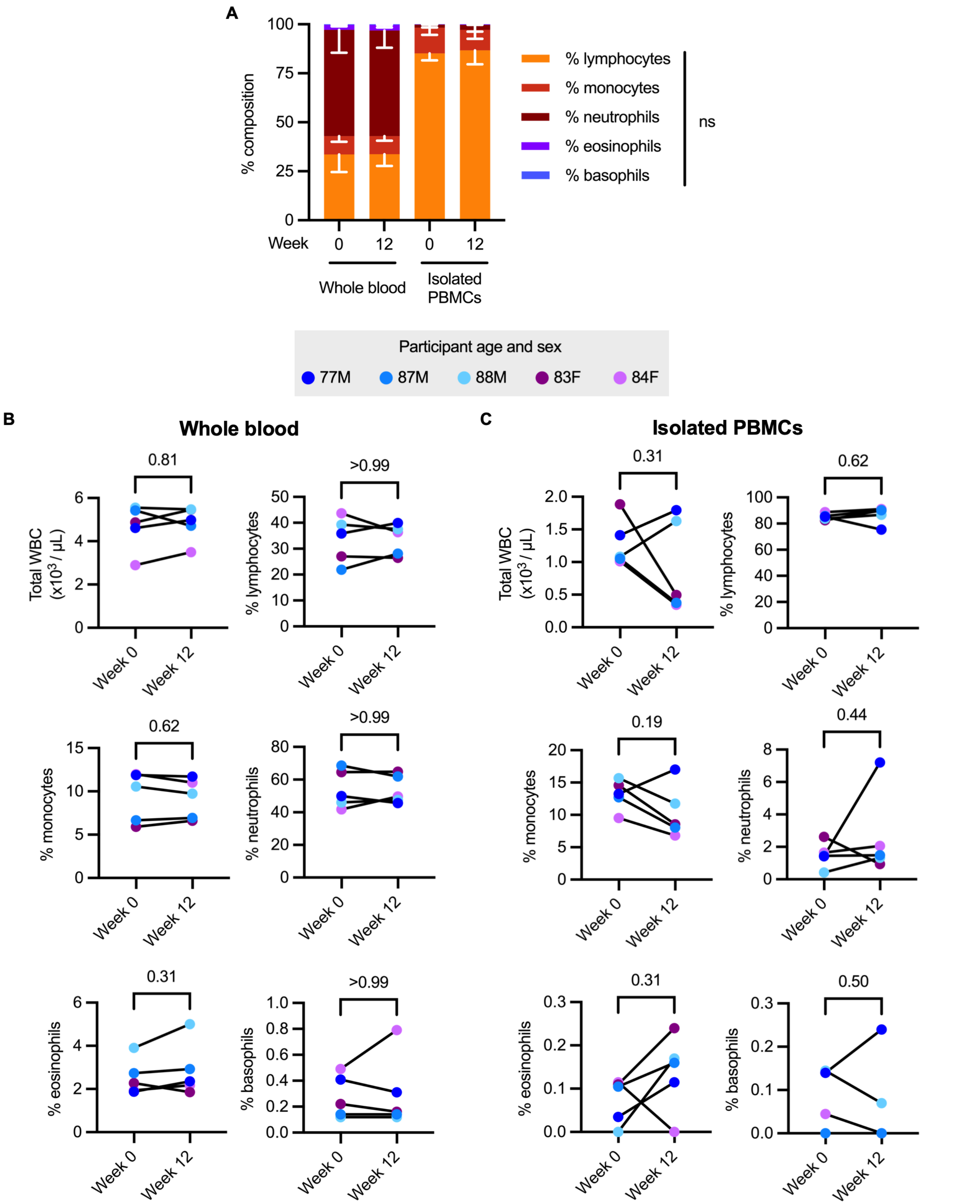

Figure S10. PBMC composition in whole blood and isolated PBMCs is consistent in STRONG participant samples from week 0 to week 12.
(**A**) PBMC composition in whole blood and isolated PBMC samples from SHOCK cohort participants on week 0 and week 12 of the exercise intervention, measured by Coulter counter analysis (N = 5). Mean ± standard deviation. Statistical comparison of cell type abundance between week 0 and week 12 of exercise within whole blood or isolated PBMC samples

performed by two-way ANOVA; ns – $p>0.05$. (**B-C**) Percent composition of STRONG participant PBMC subtypes in (**B**) whole blood and (**C**) isolated PBMC samples on week 0 and week 12 of exercise. Each data point represents the mean abundance of the indicated cell type in 0 and 200 μM CQ-treated samples for each individual. P-values by paired t-tests.
